# ADA2-deficient cells exhibit increased levels of cell death and metabolic disturbances

**DOI:** 10.1038/s41420-026-03027-9

**Published:** 2026-03-23

**Authors:** Lisa Ehlers, Marjon Wouters, Bethany Pillay, Selket Delafontaine, Giorgia Bucciol, Marco Baggio, Mariia Dzhus, Anneleen Hombrouck, Alexandra Damerau, Lien De Somer, Rik Schrijvers, Steven Vanderschueren, Maarten Jacquemyn, Tilmann Kallinich, Dirk Daelemans, Bart Ghesquière, Patrizia Agostinis, Leen Moens, Isabelle Meyts

**Affiliations:** 1 Department of Microbiology, Immunology and Transplantation, Laboratory for Inborn Errors of Immunity, KU Leuven, Leuven, Belgium; 2 Department of Pediatric Respiratory Medicine, Immunology and Critical Care Medicine, Charité – Universitätsmedizin Berlin, corporate member of Freie Universität Berlin and Humboldt-Universität zu Berlin, Berlin, Germany; 3 Berlin Institute of Health at Charité – Universitätsmedizin Berlin, Berlin, Germany; 4 German Center for Child and Adolescent Health (DZKJ), partner site Berlin, Berlin, Germany; 5 Deutsches Rheuma-Forschungszentrum, an Institute of the Leibniz Association, Berlin, Germany; 6 Department of Pediatrics, University Hospitals Leuven, Leuven, Belgium; 7 Laboratory of Computational and Developmental Biology, Berlin Institute for Medical Systems Biology (BIMSB), Max-Delbrück-Centrum for Molecular Medicine in the Helmholtz Association (MDC), Berlin, Germany; 8 Charité – Universitätsmedizin Berlin, corporate member of Freie Universität Berlin and Humboldt-Universität zu Berlin, Department of Rheumatology and Clinical Immunology, Berlin, Germany; 9 Laboratory of Immunobiology, Department of Microbiology and Immunology, KU Leuven, Leuven, Belgium; 10 Department of Microbiology, Immunology and Transplantation, Allergy and Clinical immunology Research group, KU Leuven, Leuven, Belgium; 11 Department of General Internal Medicine, University Hospitals Leuven, Leuven, Belgium; 12 Department of Microbiology, Immunology and Transplantation, KU Leuven, Leuven, Belgium; 13 European Reference Network for Immunodeficiency, Autoinflammatory, Autoimmune and Pediatric Rheumatic disease (ERN-RITA), Leuven, Belgium; 14 KU Leuven Department of Microbiology, Immunology and Transplantation, Molecular Genetics and Therapeutics in Virology and Oncology Research Group, Rega Institute for Medical Research, Leuven, Belgium; 15 Department of Cellular and Molecular Medicine, Laboratory of Applied Mass Spectrometry, KU Leuven, Leuven, Belgium; 16 Cell Death Research & Therapy (CDRT) Lab, Department of Cellular & Molecular Medicine, Center for Cancer Biology, VIB-KU Leuven, Leuven, Belgium

**Keywords:** Inflammatory diseases, Immune cell death

## Abstract

Deficiency of adenosine deaminase 2 (DADA2) causes a complex phenotype of autoinflammation and immunodeficiency. Bone marrow failure is often refractory to treatment with tumour necrosis factor-alpha (TNF-alpha) inhibitors and additional treatment options are needed. However, the pathomechanisms underlying the disease remain incompletely understood. The aim of this study was to examine the viability and metabolic profile of ADA2-deficient cells and to characterise the activity of different cell death pathways to advance the mechanistic understanding of DADA2. By flow cytometry and western blot, we showed that ADA2^-/-^ U-937 cells and PBMCs from DADA2 patients showed significantly elevated levels of cell death compared with cells expressing wild-type *ADA2*. Viability of ADA2-deficient cells was not improved by inhibitors of apoptosis, necroptosis, pyroptosis and ferroptosis. Blocking of TNF-alpha, type I interferon and STING signalling as well as reintroduction of wild-type ADA2 protein did not rescue the cell death phenotype in vitro. ADA2-deficient cells had an aberrant morphology with increased cell size and granularity and were impaired in their proliferative capacity. To identify the cause of the impaired viability, we performed ^13^C glucose tracer metabolomics experiments which revealed disturbances in the pentose phosphate pathway of ADA2-deficient cells. This tended to be associated with increased exposure to intracellular reactive oxygen species that was attenuated in the PBMCs of a DADA2 patient measured after successful hematopoietic stem cell transplantation. Collectively, our findings established increased levels of cell death as a possible pathomechanism of DADA2 and showed that the absence of ADA2 leads to an impairment of the pentose phosphate pathway which may account for the cellular vulnerability of ADA2-deficient cells.

## Introduction

Deficiency of adenosine deaminase 2 (DADA2) is an inborn error of immunity that manifests with a complex phenotype of inflammation, vasculitis, immunodeficiency, and bone marrow failure [1]. Biallelic mutations in the *ADA2* gene lead to impaired secretion of the ADA2 protein and absent ADA2 enzyme activity in the serum of DADA2 patients [2, 3]. Immunologically, patients have been described to have an elevated type I and type II interferon signature and the inflammatory phenotype is often responsive to treatment with tumour necrosis factor-alpha inhibitors (TNFi) [4, 5]. DADA2 patients presenting with bone marrow failure often require hematopoietic stem cell transplantation (HSCT) and disease lethality is 8% [6]. Despite the increasing number of reported patients and studies characterising large numbers of pathogenic variants [7, 8], genotype-phenotype correlations are difficult to establish and the pathomechanisms driving the disease remain elusive [9, 10]. As a consequence, new treatment targets have hardly been identified since the initial description of the disease. Consequently, a better grasp of the pathways driving the disease is urgently needed.

Early studies into the mechanisms underlying DADA2 mentioned the increased propensity of ADA2-deficient cells for cell death or membrane instability [2, 11]. Regulated cell death (RCD) has been established as a driver of inflammation and damage or danger signal, and cytokines can in turn induce inflammatory forms of cell death [12].

Therefore, we analyse levels and different forms of cell death in primary human immune cells from healthy controls (HC) and DADA2 patients and ADA2-deficient U-937 cells and describe the metabolic profile of DADA2 patients.

## Results

### Peripheral blood mononuclear cells from DADA2 patients show increased levels of basal cell death but reduced induction of necroptosis in vitro

**Fig. 1:**
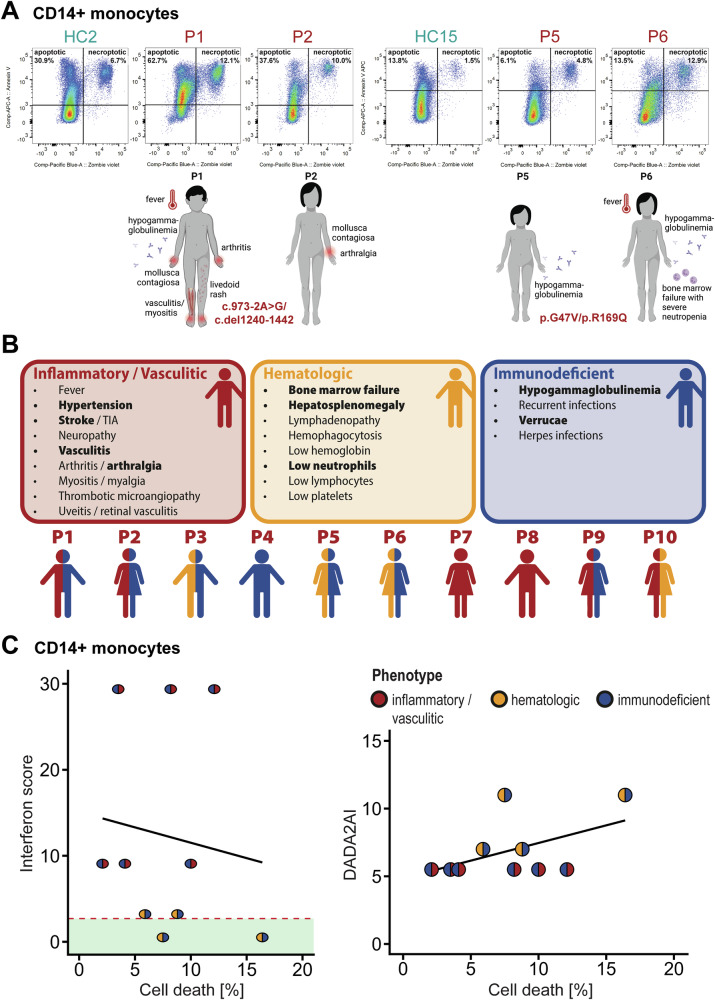
Phenotype and cell death levels in DADA2 patients. **A** Cell death of healthy control (HC) and DADA2 (P) CD14+ monocytes was measured after 5 h incubation in vitro by flow cytometry. Dead cells were identified by staining with Annexin V and Zombie® viability dye. The bottom part of the figure depicts the clinical phenotype of the patients at the time of sampling. Patients P1 and P2 as well as P5 and P6 are siblings. **B** Symptoms captured by the DADA2 activity index (DADA2AI) are displayed assigned to the corresponding disease phenotype [13, 14]. Below, the predominant phenotype of the included patients is indicated by colour (red = inflammatory / vasculitic, yellow = haematologic, blue = immunodeficient). Detailed information is provided in Supplementary Table S1. **C** Correlation of type I interferon signature in whole blood (*left panel*) or disease activity measured by DADA2AI (*right panel*) with cell death levels of CD14+ monocytes from healthy controls (HC) and DADA2 patients measured by flow cytometric measurement of staining with Annexin V and Zombie® viability dye. The interferon score was determined by qPCR as described by Rice et al. [36]. The green band indicates the normal reference range.

We isolated mononuclear cells (PBMCs) from the peripheral blood of ten DADA2 patients (Supplementary Table S1) and analysed their viability compared with PBMCs from healthy donors. Loss of viability was determined by flow cytometry after 5 h monoculture of isolated CD14+ monocytes. We found that baseline levels of cell death were significantly higher in the DADA2 patients’ cells (7.59 ± 1.11% [mean ± standard error of the mean] compared with HC (3.87 ± 0.43%). In two pairs of siblings with identical genotypes but different clinical phenotypes, the severity of their clinical symptoms correlated with the levels of cell death at baseline (Fig. 1A). To further elucidate the relationship between the clinical phenotype and cell death, we characterised our cohort with respect to the patients’ predominant phenotype as defined in the DADA2 management guidelines [13]. For patients with ample clinical information, the DADA2 disease activity index (DADA2AI) was determined [14]. Fig. 1B shows the leading symptoms of the respective disease phenotypes reflected within the DADA2AI. In a subcohort of our patients, cell death levels in CD14+ monocytes weakly correlated with disease activity (r = 0.5, *p* = 0.14) (Fig. 1C). This tendency was not observed when evaluating whole blood interferon scores (r = -0.1, *p* = 0.73) (Fig. 1C). We did however find a higher interferon signature in DADA2 patients with an inflammatory phenotype compared with those with predominant bone marrow failure irrespective of disease activity (Fig. 1C).

In addition, we analysed levels of cyclophilin A in the urine of our patients compared to paediatric HC as a marker of necroptotic cell death [15]. Here, we did not observe an increase in cyclophilin A (Supplementary Fig. S1). Urine samples were however collected in the flare-free interval in all patients.

**Fig. 2:**
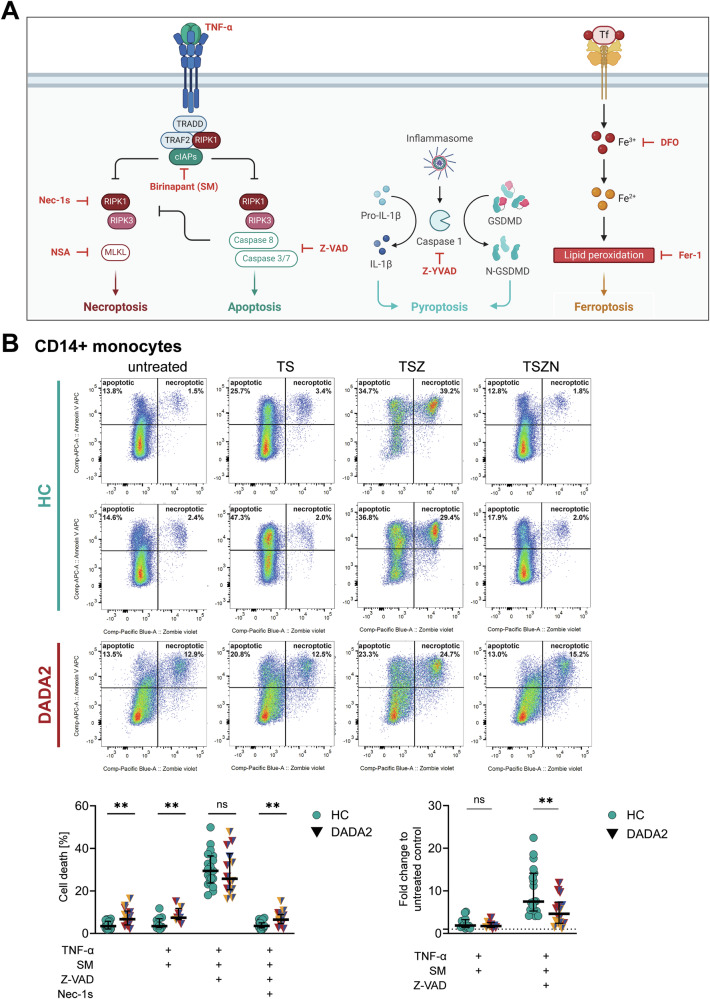
Analysis of cell death in DADA2 at baseline and after induction of apoptosis and necroptosis in vitro. **A** Schematic overview of the induction and inhibition of different regulated cell death pathways in vitro. **B** Cell death in CD14+ monocytes from healthy controls (HC) and DADA2 patients was measured by flow cytometric measurement of staining with Annexin V and Zombie® viability dye. Cells were incubated for 5 h. Apoptosis (TS) and necroptosis (TSZ) were induced by stimulation with 100 nM birinapant (SM) ± 20 µM Z-VAD-FMK (Z-VAD) followed by 20 ng/mL tumour necrosis factor-alpha (TNF-α). Inhibition of necroptosis was achieved by adding 10 µM necrostatin-1s (Nec-1s, TSZN). Plots show median and 25 and 75th percentiles. Data from *n* = 19 (HC) and *n* = 14 (DADA2, comprising *n* = 7 distinct patients) independent experiments are shown. Mann-Whitney-U test, **p < 0.01. The patients’ phenotype is indicated by colour as displayed in Fig. 1B. *Legend:* cIAPs cellular inhibitor of apoptosis proteins, DFO deferoxamine, Fer-1 ferrostatin-1, GSDMD gasdermin D, IL-1β interleukin-1beta, MLKL mixed lineage kinase domain like pseudokinase, Nec-1s necrostatin-1s, NSA necrosulfonamide, RIPK1 receptor-interacting serine/threonine-protein kinase 1, RIPK3 receptor-interacting serine/threonine-protein kinase 3, SM Smac mimetic, TRADD tumour necrosis factor receptor type 1-associated DEATH domain protein, TRAF2 TNF receptor-associated factor 2, Z-VAD Z-VAD-FMK, Z-YVAD, Z-YVAD-FMK.

We hypothesised that the difference in cellular viability was due to a higher baseline activation of inflammatory pathways in DADA2 which could further sensitise these immune cells to RCD. To test this hypothesis, we induced necroptosis as well as apoptosis in vitro in HC and DADA2 monocytes as RCD paradigms with opposite inflammatory outputs. The Smac mimetic (SM) birinapant promotes degradation of cellular inhibitor of apoptosis proteins (cIAPs), thereby inducing apoptosis upon stimulation with TNF-alpha in vitro. Simultaneous inhibition of caspases by Z-VAD-FMK (Z-VAD) blocks apoptosis and causes necroptotic cell death (Fig. 2A). While exhibiting elevated levels of cell death at baseline, CD14+ monocytes of DADA2 patients did not show increased levels of apoptosis or necroptosis upon in vitro induction of inflammatory cell death (Fig. 2B). Of note, we found that there was reduced induction of necroptosis when measured by fold-change to baseline levels of cell death (Fig. 2B). Induction of apoptosis was also weaker in ADA2-deficient cells although in primary monocytes the difference was not significant (Fig. 2B). The absolute levels of cell death after necroptosis induction were however not different between HC and DADA2 cells (Fig. 2B). No relationship was found between the cell death levels and the patients’ predominant clinical phenotype (Fig. 2B).

**Fig. 3:**
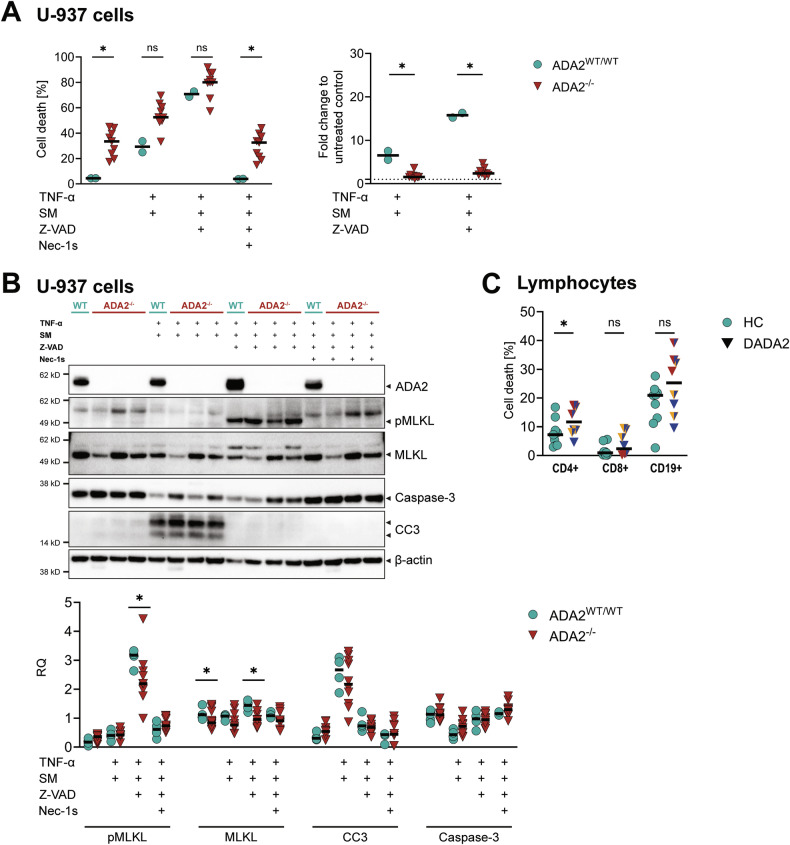
Analysis of cell death in ADA2^-/-^ U-937 cells at baseline and after induction of apoptosis and necroptosis in vitro. **A** Cell death in U-937 cells was measured by flow cytometric measurement after staining with Annexin V and Zombie® viability dye. Cells were incubated for 5 h. Apoptosis and necroptosis were induced by stimulation with 100 nM birinapant (SM) ± 20 µM Z-VAD-FMK (Z-VAD) followed by 20 ng/mL TNF-α. Inhibition of necroptosis was achieved by adding 10 µM necrostatin-1s (Nec-1s). The experiment was performed for *n* = 5 distinct ADA2^-/-^ U-937 cell clones. **B** Induction of apoptosis and necroptosis in U-937 cells by western blot. Whole cell lysates were produced after 5 h incubation as for (**A**). The plots show protein levels from *n* = 5 independent experiments. All blots are depicted in Supplementary Fig. S2C. **C** Cell death in lymphocytes from healthy controls (HC) and DADA2 patients was measured by flow cytometry after staining with Annexin V and Zombie® viability dye after 48-hour incubation in vitro. Data from *n* = 9 (HC) and *n* = 8 (DADA2, comprising *n* = 7 distinct patients) independent experiments are shown. The median is indicated in all plots. Mann-Whitney-U test, *p < 0.05. The patients’ phenotype is indicated by colour as displayed in Fig. 1B. *Legend:* CC3 cleaved caspase-3, MLKL mixed lineage kinase domain like pseudokinase, Nec-1s necrostatin-1s, SM Smac mimetic, Z-VAD Z-VAD-FMK.

Both the increase in baseline cell death and the attenuated necroptosis induction were confirmed in ADA2^-/-^ U-937 cell lines (Fig. 3A). Apoptosis and necroptosis induction were verified by cleavage of caspase-3 and phosphorylation of mixed lineage kinase domain like pseudokinase (MLKL) and receptor-interacting serine/threonine-protein kinase 1 (RIPK1) on western blot (Fig. 3B, Supplementary Fig. S2B, C). In line with our findings, we showed reduced levels of phosphorylated MLKL in ADA2^-/-^ U-937 cells after induction of necroptosis in vitro compared with cells expressing wild-type ADA2 (Fig. 3B). Levels of total MLKL were lower both at baseline and after necroptosis induction (Fig. 3B). No cleavage of caspase-3 was observed upon necroptosis induction.

To determine whether the increase in cell death levels in DADA2 patients’ cells persisted outside the context of the inflammatory milieu present in vivo, we analysed cell death after increasing incubation times in vitro. These experiments were performed in lymphocytes because of their longer lifespan in culture. After 48 h, CD4 + T cells showed significantly increased levels of cell death in DADA2 compared to HC. A tendency was also observed in CD8+ and CD19+ lymphocytes (Fig. 3C). Again, patients with the highest levels of cell death showed varying phenotypes (Fig. 3C).

In summary, we showed that PBMCs from DADA2 patients as well as ADA2^-/-^ U-937 cells exhibit elevated levels of cell death at baseline but a restrained response to apoptosis and necroptosis induction in vitro. This phenotype tended to correlate with disease activity but was independent of the leading disease phenotype.

### Increased levels of cell death in DADA2 are not rescued by inhibitors of RCD

**Fig. 4:**
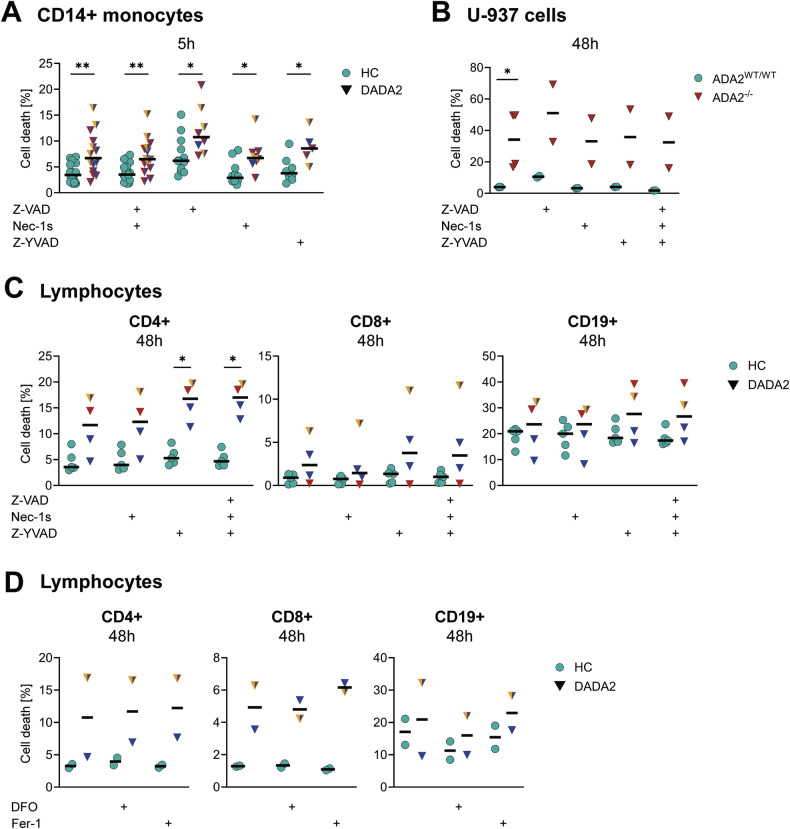
Analysis of cell death in DADA2 after inhibition of regulated cell death pathways. Cell death was measured by flow cytometric measurement of staining with Annexin V and Zombie® viability dye. Apoptosis, necroptosis and pyroptosis were inhibited by incubation with 20 µM Z-VAD-FMK (Z-VAD), 10 µM necrostatin-1s (Nec-1s) and 20 µM Z-YVAD-FMK (Z-YVAD), respectively, for the indicated periods of time. **A** Data from *n* = 12 | 12 | 8 (HC) and *n* = 8 | 8 | 5 (DADA2) independent experiments for Z-VAD|Nec-1s|Z-YVAD, respectively, are shown. **B** The experiment was performed for *n* = 2 distinct ADA2^-/-^ U-937 cell clones. **C** Data from *n* = 5 (HC) and *n* = 4 (DADA2) independent experiments are shown. **D** Ferroptosis was inhibited by 48-hour incubation with 50 µM deferoxamine (DFO) or 2 µM ferrostatin-1 (Fer-1) in *n* = 2 HC and DADA2 patients. The median is indicated in all plots. Mann-Whitney-U test, *p < 0.05, **p < 0.01. The patients’ phenotype is indicated by colour as displayed in Fig. 1B.

We then sought to evaluate whether the increased levels of spontaneous cell death of DADA2 cells were due to a baseline activation of RCD pathways. We therefore incubated control and ADA2-deficient cells with inhibitors of apoptosis (Z-VAD, pan-caspase inhibitor), necroptosis (necrostatin-1s, inhibitor of RIPK1) and pyroptosis (Z-YVAD, inhibitor of caspase-1) (Fig. 2A). Efficacy of the inhibitors was confirmed by successful suppression of apoptosis and necroptosis induction as shown by western blot (Fig. 3B, Supplementary Fig. S2B, C). The inhibitors did not improve viability of DADA2 CD14+ monocytes (Fig. 4A). These findings were confirmed after 48 h incubation of ADA2-deficient U-937 cells as well as primary lymphocytes with the RCD inhibitors (Fig. 4B, C). We concluded that the increase in baseline cell death observed in ADA2-deficient cells was not due to an upregulation of apoptosis, necroptosis or pyroptosis. Ferroptosis is another form of RCD driven by iron-mediated lipid peroxidation linked to inflammation [16]. Elevated levels of cell death in lymphocytes from DADA2 patients also persisted after 48 h incubation with the ferroptosis inhibitors deferoxamine (DFO) and ferrostatin-1 (Fer-1) (Fig. 4D).

In conclusion, we showed that inhibition of major forms of RCD pathways does not rescue the spontaneous cell death phenotype of ADA2-deficient cells.

### Cell death is not induced extrinsically by the “DADA2 milieu”

**Fig. 5:**
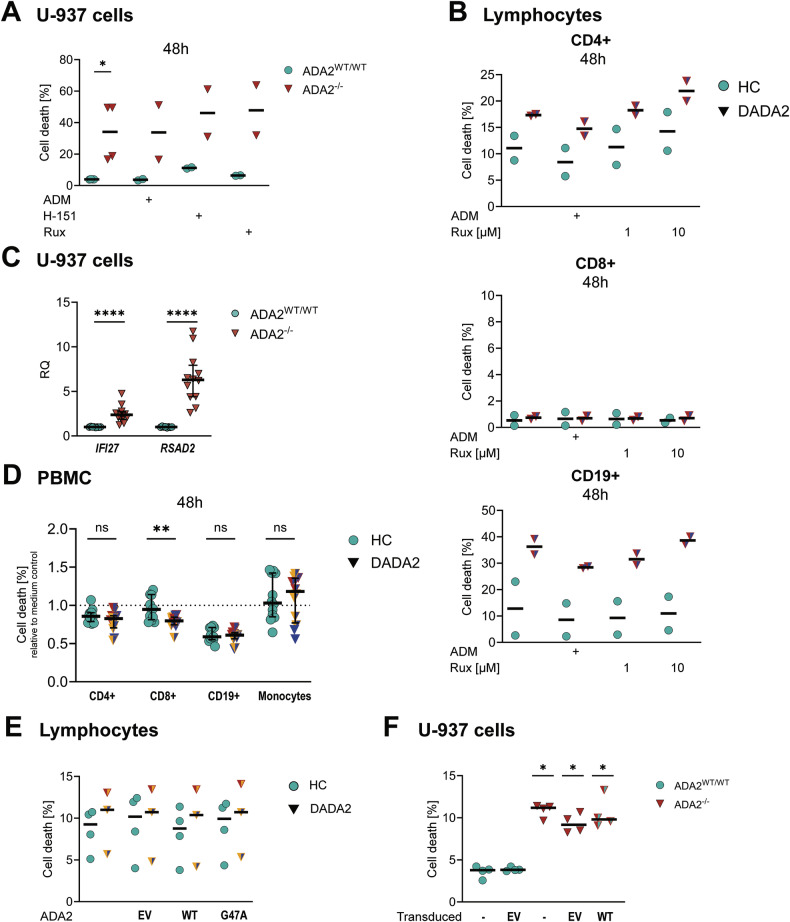
Cell death in DADA2 upon inhibition of inflammatory pathways. Cell death was measured by flow cytometric measurement of staining with Annexin V and Zombie® viability dye in (**A**) U-937 cells and (**B**) lymphocytes after incubation with 5 μg/mL adalimumab (ADM), 5 µM ruxolitinib (Rux) or 1 µM H-151 for the indicated periods of time. **C** Gene expression of the interferon stimulated genes *IFI27* and *RSAD2* was determined by qPCR in ADA2^WT/WT^ and ADA2^-/-^ U-937 cells. The experiment was performed for *n* = 5 distinct ADA2^-/-^ U-937 cell clones measured at multiple time points. mRNA expression was normalised to *HPRT1* and is depicted relative to the WT-expressing cells (RQ). **D** PBMCs from two healthy controls were incubated with supernatant of PBMCs from healthy controls (HC) (*n* = 6) and DADA2 patients (*n* = 6) for 48 h. Cell death was measured by flow cytometric measurement of staining with Annexin V and Zombie® viability dye. **E** Cell death in lymphocytes from healthy controls (HC) and DADA2 patients was measured by flow cytometry after 48 h incubation with supernatant of HEK293T cells overexpressing the indicated *ADA2* variants. Cell death was determined by staining with Annexin V and Zombie® viability dye. **F** Cell death in ADA2^WT/WT^ and ADA2^-/-^ U-937 cells was measured by flow cytometry after transduction with empty vector or WT *ADA2* to restore ADA2 protein expression (Supplementary Fig. S4). Significance is depicted as compared to cell death levels of ADA2^WT/WT^ cells transduced with EV. All plots show median and 25 and 75th percentiles where applicable. Mann-Whitney-U test, *p < 0.05, **p < 0.01, ****p < 0.0001. The patients’ phenotype is indicated by colour as displayed in Fig. 1B. *Legend:* EV empty vector, WT wild type.

As many DADA2 patients are effectively treated with TNFi, we next examined whether this treatment attenuated the cell death phenotype of ADA2-deficient cells in vitro. Efficacy of the TNFi adalimumab in rescuing TNF-alpha induced cell death in vitro was verified by flow cytometry (Supplementary Fig. S2A). Incubation with adalimumab over 48 hours did not affect the levels of cell death in ADA2-deficient U-937 cells or DADA2 lymphocytes (Fig. 5A, B). As DADA2 cells exhibit an increased type I interferon signature (Fig. 5C) and type I interferons have been shown to induce PANoptosis [17], we hypothesised that inhibition of JAK1/2 signalling by ruxolitinib might attenuate cell death in DADA2. As for adalimumab, 48 h incubation with ruxolitinib did not improve viability (Fig. 5A, B). Based on previous findings suggesting that the inflammatory phenotype of ADA2-deficient cells is mediated by intracellular DNA-sensing through STING [18], we also explored the effect of STING inhibition by H-151 on cell death in ADA2-deficient U-937 cells and did not find an improvement (Fig. 5A). Efficacy of ruxolitinib and H-151 in suppressing the type I interferon response was verified in vitro (Supplementary Fig. S3).

To further test the hypothesis that the increase in cell death in DADA2 is caused extrinsically by the “secretome” of ADA2-deficient cells, we collected supernatants of PBMCs from HC and DADA2 patients after 48 h culture in vitro. Incubation of HC PBMCs with supernatants from DADA2 cells for another 48 h did not cause increased levels of cell death compared with incubation with supernatant from HC PBMCs (Fig. 5D).

Finally, lymphocytes were incubated with supernatant of HEK293T cells overexpressing WT and mutant (p.G47A) ADA2 protein—a variant exhibiting residual ADA2 secretion [19]. Extrinsic addition of functional ADA2 did not improve viability of ADA2-deficient cells (Fig. 5E). Similarly, transducing ADA2^-/-^ U-937 with WT *ADA2* did not rescue the cell death phenotype despite restoring physiological levels of ADA2 protein expression (Fig. 5F, Supplementary Fig. S4).

Collectively, our data suggest that the increased susceptibility to undergo spontaneous cell death in DADA2 is cell-intrinsic and not mediated by the inflammatory milieu of ADA2-deficient cells and cannot be rescued by inhibition of TNF-alpha, type I interferon signalling or STING signalling.

### ADA2-deficient cells exhibit metabolic disturbances

**Fig. 6:**
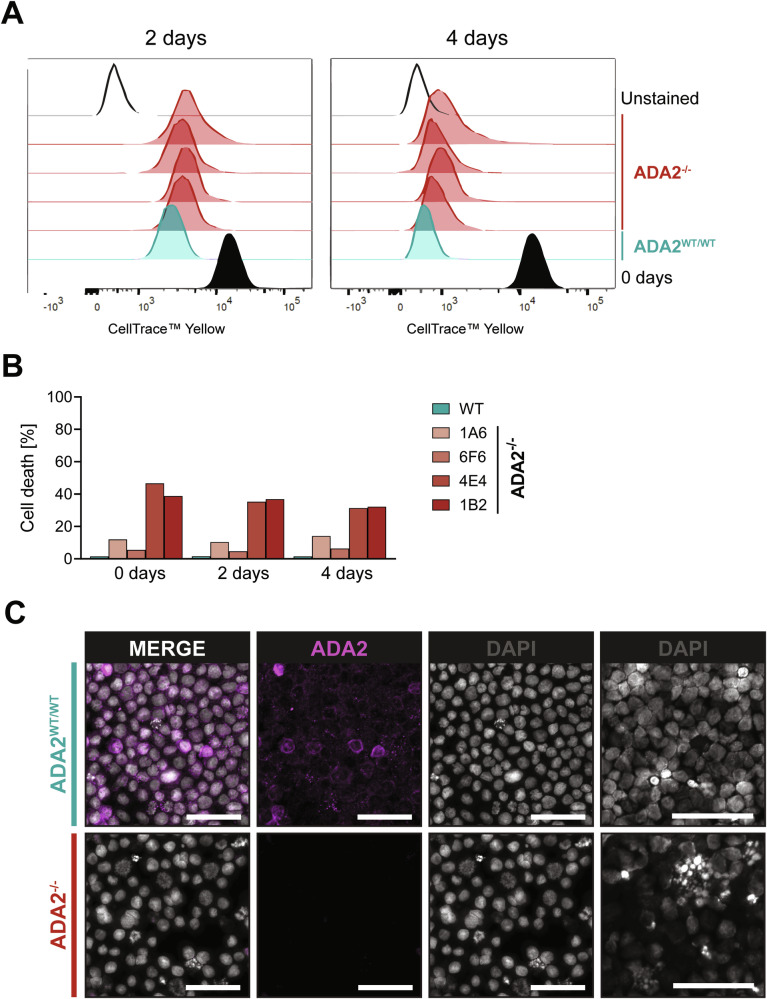
Proliferation and morphology of ADA2-deficient cells. **A** CellTrace™ Proliferation Assay was performed in ADA2^WT/WT^ and ADA2^-/-^ U-937 cells over 4 days. Cells were prepared for flow cytometry at baseline and after 2 and 4 days of incubation. **B** Cell death of ADA2^WT/WT^ and four different clones of ADA2^-/-^ U-937 cells was measured by flow cytometry during the incubation of the CellTrace™ Proliferation Assay. Dead cells were identified as Annexin V / Zombie® double-positive cells. **C** Immunofluorescence microscopy of ADA2^WT/WT^ and ADA2^-/-^ U-937 cells stained for ADA2. DAPI was used for nuclear staining. The scale bar represents 50 µm.

**Fig. 7:**
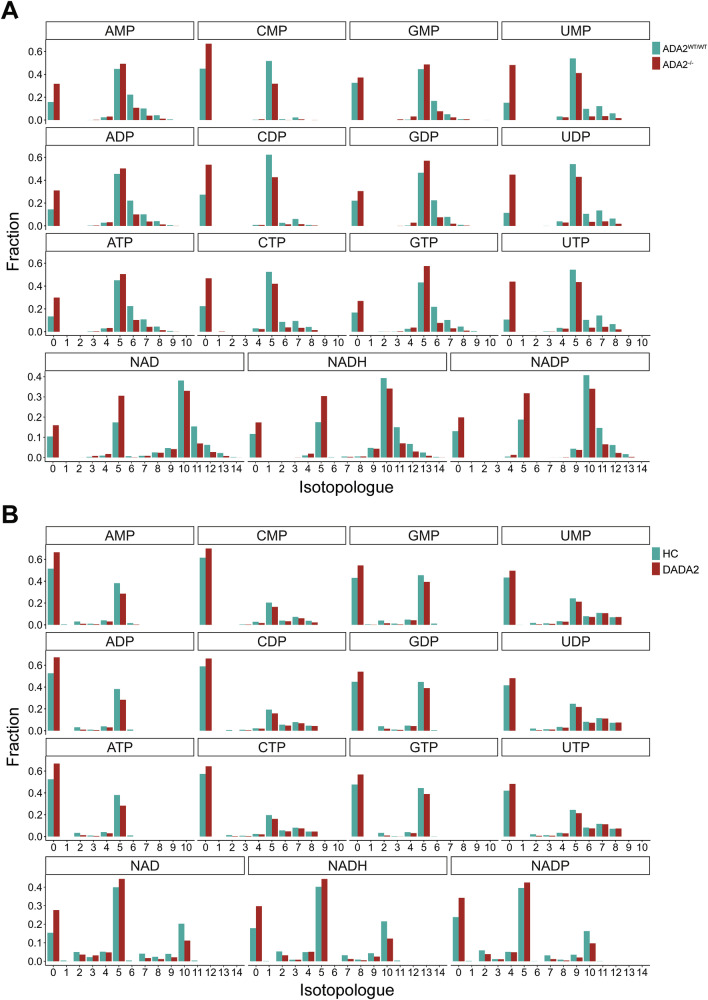
Metabolomics of ADA2-deficient cells. **A** Tracer metabolomics after 24 h incubation in RPMI medium containing ^13^C-glucose. The analysis shows ADA2^WT/WT^ (*n* = 1) and ADA2^-/-^ (*n* = 3) U-937 cells. **B** Tracer metabolomics were performed in healthy control (HC) (*n* = 1) and DADA2 (*n* = 1) human monocyte-derived macrophages after 24 h incubation in RPMI medium containing ^13^C-glucose. The bar graphs show the average fraction of isotopologues over the three replicates. The x-axis indicates the number of labelled C atoms in the respective molecule.

**Fig. 8:**
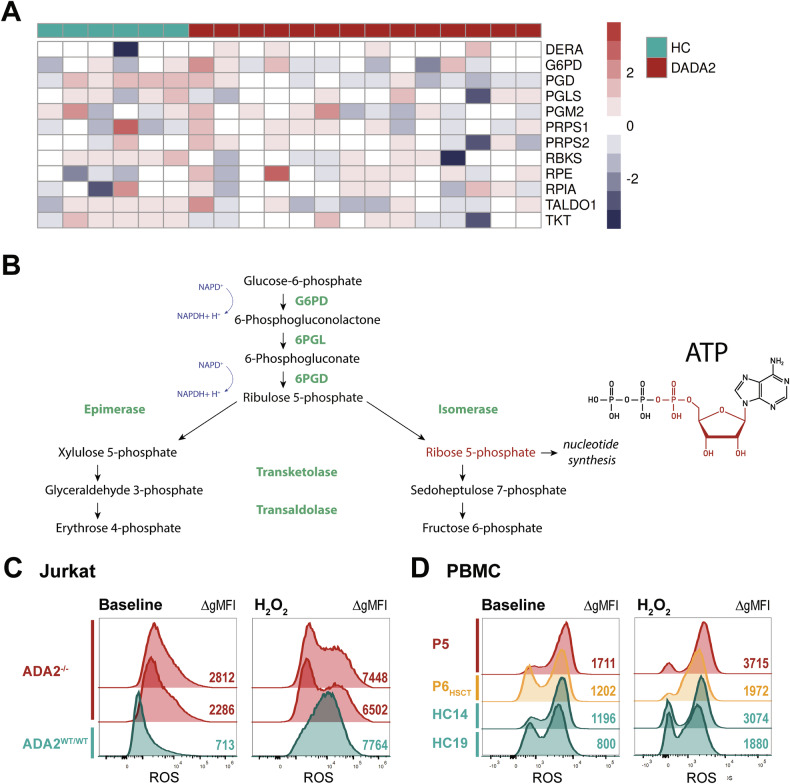
Pentose phosphate pathway and oxidative stress in DADA2. **A** Analysis of gene expression by single-cell RNA sequencing in HC and DADA2 monocytes based on the data published by Watanabe et al. (GSE142444) [11]. Heatmap showing the normalised log reads (z-scores) of the genes associated with the pentose phosphate pathway (reactome id R-HSA-71336). Of these, *PGD* and *RBKS* show a significant difference at logFC = −0.37, FDR = 0.03 and logFC = −2.35, FDR = 0.02 respectively. **B** Schematic overview of the reactions of the pentose phosphate pathway. **C** Total content of reactive oxygen species was measured by flow cytometry in ADA2^WT/WT^ and ADA2^-/-^ Jurkat cells at baseline and after incubation with H_2_O_2_. **D** Total content of reactive oxygen species was measured by flow cytometry in PBMCs from healthy controls (HC) and DADA2 patients P5 and P6 at baseline and after incubation with H_2_O_2_. P6 had undergone successful HSCT at the time of sampling. *Legend:* 6PGD 6-phosphogluconate dehydrogenase, 6PGL 6-phosphogluconolactonase, G6PD glucose-6-phosphate dehydrogenase, gMFI geometric mean fluorescence intensity, ROS reactive oxygen species.

Since we observed that ADA2-deficient U-937 cells show a substantially reduced cell growth in vitro compared with wild-type ADA2-expressing cells, we hypothesised that these cells exhibit impaired cell proliferation. By CellTrace™ Proliferation Assay, we identified a slight reduction in cell proliferation in ADA2^-/-^ U-937 cells (Fig. 6A). In the same experiment, we did however identify increased levels of cell death as the main contributor to the reduced cell growth over the observation period (Fig. 6B). By microscopy, we observed an aberrant morphology of the ADA2^-/-^ U-937 cell clones with a prominent increase in cell size (Fig. 6C, Supplementary Fig. S5). As cell size has been linked to changes in metabolism [20, 21], we hypothesised that metabolic disturbances contribute to the increase in spontaneous cell death in ADA2 deficiency. We performed tracer metabolomics experiments with ^13^C-labelled glucose-containing medium in wild-type and ADA2^-/-^ U-937 cells (Fig. 7A). We found that ADA2-deficient cells showed decreased labelling of metabolites like NMP/NDP/NTPs, NAD(H), Acetyl (CoA) and UDP-GlcNAc that are built from nucleotides containing a ribose-5-phosphate group (Fig. 7A). These findings indicate an impairment of the pentose phosphate pathway. Many of these metabolites were also significantly reduced in overall abundance in ADA2-deficient cells (Supplementary Fig. S6). As proof of concept, we performed another tracer metabolomics experiment in monocyte-derived macrophages from a DADA2 patient compared with a HC. DADA2 cells showed the same reduction in labelling of metabolites synthesised with ribose-5-phosphate from the pentose phosphate pathway (Fig. 7B) and the same decrease in their overall abundance (Supplementary Fig. S7). To further validate our findings, we analysed previously published single-cell RNA sequencing data from monocytes of HCs and DADA2 patients [11]. In line with the results of the metabolomics experiments, there was a significant decrease in *PGD* expression in DADA2 monocytes (Fig. 8A). *PGD* encodes 6-phosphogluconate dehydrogenase (6PGD), one of the key enzymes of the pentose phosphate pathway (Fig. 8B). A critical function of the pentose phosphate pathway is the production of NADPH + H^+^ for the regeneration of glutathione [22]. We therefore hypothesised that the impairment of this pathway might affect antioxidant capacity. We found that ADA2-deficient Jurkat cells showed elevated levels of reactive oxygen species (ROS) compared with the WT-expressing cell line (Fig. 8C). Exposure to extrinsic oxidative stress by H_2_O_2_ attenuated these differences (Fig. 8C). We confirmed this finding in the PBMCs of DADA2 patient P5 (Fig. 8D). As proof of principle, we also included a sample from patient P6 that had at this point undergone successful HSCT (Fig. 8D). Indeed, we found baseline ROS levels close to HC in the transplanted patient’s PBMCs, hinting at a rescue of the metabolic phenotype upon HSCT in line with the clinical resolution of the patient’s phenotype (Fig. 8D).

Overall, we showed that ADA2-deficient cells exhibit an impairment in the pentose phosphate pathway likely leading to an impairment of the oxidative stress response that is improved by HSCT.

## Discussion

In this study, we show that ADA2 deficiency is characterised by increased levels of cell death and an impairment of the pentose phosphate pathway.

The observation that ADA2-deficient cells are more prone to spontaneous cell death was already reported in the first description of the disease [2]. Additional reports remarked on the fragility of DADA2 patients’ cells in culture [11]. Despite these findings, the mechanisms underlying the increased levels of cell death and its contribution to the immunological and clinical phenotype of DADA2 remained elusive. Recently, the role of inflammatory cell death in the pathogenesis of inflammatory diseases has been increasingly explored [15, 23, 24]. The advent of targeted therapies directed against key cytokines involved in the different cell death pathways highlights the clinical importance of identifying the leading pathways to guide treatment choices: Patients whose phenotype is driven by TNF-alpha-induced necroptotic cell death may benefit from TNFi, and interleukin-1 inhibitors have markedly improved the outcome of patients with autoinflammatory diseases driven by increased pyroptosis [23, 25].

Especially DADA2 patients whose predominant clinical phenotype is vasculitis clinically benefit from treatment with TNFi. We therefore suspected an involvement of necroptosis in the inflammatory phenotype of the disease. While our data confirm that elevated levels of baseline cell death are a characteristic of ADA2-deficient cells, the phenotype is unresponsive to inhibitors of the necroptotic pathway. Our findings suggest that cell death in DADA2 is not driven by an RCD pathway (i.e., apoptosis, necroptosis, pyroptosis or ferroptosis). On the contrary, the elevated baseline levels of cell death appear to impair the ability of ADA2-deficient cells to undergo necroptosis (Figs. 2, 3). Cells expressing pathogenic variants of *ADA2* have been reported to exhibit an increased ER stress response [7]. A severe ER stress response is classically associated with an increase in apoptosis [26]. In our experiments, the cell death phenotype of ADA2-deficient cells did not resolve upon pharmaceutical inhibition of apoptosis.

Importantly, in vitro treatment with TNFi did not attenuate the cell death phenotype. TNFi have previously been shown to rescue some of the immunological phenotype of ADA2-deficient immune cells [5]. All patients included in our study were on treatment with TNFi. The cell death phenotype was present regardless of the ongoing immunomodulatory therapy, suggesting that aberrant TNF signalling is not the cause of cell death in DADA2. Similarly, Janus kinase and STING inhibitors did not rescue the phenotype, indicating that increased type I interferon and STING-signalling are not sufficient to cause increased cell death in isolation. Taking these findings into account, we conclude that inhibition of inflammation does not prevent cell death in ADA2-deficient cells and hypothesise that the two phenotypes may emerge independently. Likewise, we also analysed cell death considering the patients’ predominant disease phenotypes. Consistent with the inhibitor experiments, we did not observe increased cell death in PBMCs of patients with an inflammatory/vasculitic phenotype compared with those with predominant bone marrow failure. Overall, disease phenotypes were distributed equally across samples with more or less prominent cell death levels. However, we found that disease severity tended to correlate with the severity of the cell death phenotype. This was particularly evident in two pairs of siblings with diverging disease severity and corresponding cell death levels. Our attempt to objectify this finding using the DADA2AI only showed a mild association. This is likely due to the small sample size and the fact that the activity index was designed with the predominant goal to capture disease activity in patients over time, noting improvement or worsening of symptoms [14]. Its design is therefore not ideal for the inter-patient comparison performed in this study. Our data do however suggest that the cellular mechanisms that enhance cell death in ADA2-deficient cells contribute to the severity of the clinical phenotype.

DADA2 patients with a predominant phenotype of bone marrow failure are often unresponsive to treatment with TNFi and the pathomechanism driving cytopenia in DADA2 is not yet understood [6]. Our results indicate that—independently of TNF-alpha signalling—increased cell death may contribute to bone marrow failure in DADA2. At present, DADA2 patients with treatment-refractory cytopenia often require HSCT and additional therapeutic options are needed. Identifying the causes of cell death in DADA2 will be crucial to improve the treatment of these patients.

ADA2 was initially characterised as a secreted homodimeric enzyme that mediates adenosine deamination in the extracellular space. Due to its weak affinity to adenosine and the superior catalytic activity of its isoenzyme ADA1, recent research has suggested an intracellular deaminase-independent function of ADA2 [18, 27, 28]. Since the cell death phenotype was not reproduced in HC PBMCs by medium transfer from DADA2 cells, our data indicate that the mechanisms that affect viability of ADA2-deficient cells are cell-intrinsic. A limitation to this experiment is that the supernatants from DADA2 cells likely do not perfectly mimic the extracellular environment found in vivo. In addition, the cell death phenotype was not rescued by reintroducing ADA2 to ADA2-deficient cells in vitro. This could indicate that cells exhibit long-term homoeostatic dysregulation, e.g. due to epigenetic changes, induced by the prior absence of ADA2. It is therefore likely that ADA2 deficiency causes increased cell death via an indirect route that is not immediately fixed by adding functional ADA2. Considering the uncertainty regarding the physiologically relevant cellular function of ADA2 [10], we will need more in depth studies to identify the mechanistic relationship between absence of ADA2 and cellular disturbances. The proposed indirect mechanism is in line with the fact that increased senescence has also been observed in mesenchymal stromal cells of DADA2 patients – cells with minimal endogenous ADA2 protein expression [29].

Until now, the focus of DADA2 research has been on the inflammatory processes underlying the disease [2, 11, 30]. By identifying increased cell death as a feature of DADA2, we describe an additional mechanism that is according to our data not directly linked to the inflammatory signature. By microscopy, we observed striking differences in the cell morphology of ADA2-deficient cells. We therefore assumed that ADA2 deficiency affects pathways involved in cell structure and metabolism. The traditional function of ADA2 as a deaminase is in the purine metabolism [31]. We therefore performed tracer metabolomics experiments and identified an impairment in the pentose phosphate pathway in the absence of ADA2. Expression of *PGD* is downregulated in DADA2 monocytes (Fig. 8A), possibly accounting for the metabolic impairment we observed. Suppression of *PGD* has been found to cause ER stress and impaired protein secretion – both cellular characteristics of DADA2 [3, 7, 32]. It is therefore possible that the decrease in 6-phosphogluconate dehydrogenase links different aspects of the pathophysiology of ADA2-deficient cells. Further work will be needed to understand how ADA2 deficiency affects *PGD* expression. Adenosine—the substrate of ADA2 activity—contains a ribose group that is supplied by the pentose phosphate pathway. Nucleotides and nucleosides including adenosine have diverse cellular functions ranging from DNA building blocks over energy supply to signal transmitters. At the same time the pentose phosphate pathway is involved in cellular homoeostasis by providing NADPH + H^+^ for glutathione regeneration (Fig. 8B) [22]. Thus, a dysfunction of the pentose phosphate pathway is likely to disrupt multiple cellular functions and cause a diverse cellular phenotype, such as the one seen in DADA2. In an initial experiment, we confirmed that ADA2-deficient cells showed an increased ROS content in line with a reduced antioxidant capacity. Further studies will be needed to verify the contribution of this mechanism to the DADA2 phenotype and to evaluate its potential as a therapeutic target.

This study provides the first in-depth analysis of cell death in DADA2. The main limitation of the results presented here is that they largely convey negative findings. The study was conducted in a hypothesis-guided way. While our findings strongly indicate that cell death in DADA2 is not driven by one of the major RCD pathways, it cannot be excluded that these pathways still have a role in the pathogenesis of DADA2. RCD pathways are interconnected and difficult to analyse in isolation. It is however clear from our data that there must be additional drivers as combined specific inhibition of apoptosis, necroptosis, and pyroptosis did not resolve the primary phenotype.

Our study is the first to identify a metabolic dysfunction in ADA2-deficient cells. While the mechanistic basis of the differences is yet to be understood, these findings provide the foundation to explore a new field in ADA2 research. Given the urgent need for improved treatment options, especially in DADA2 patients with bone marrow failure, this new direction could be crucial in the search for new therapeutic targets in DADA2.

In conclusion, we demonstrate increased levels of cell death in ADA2-deficient cells that are not due to RCD pathways and establish an impaired pentose phosphate pathway as a potential cause of this phenotype.

## Materials and methods

### Human subjects and sample preparation

The patients were treated at University Hospitals Leuven and their parents were included as heterozygous carriers. Healthy donors were recruited at University Hospitals Leuven and KU Leuven. All study participants provided informed consent. A detailed description of the sample processing has been reported before and parts of the methodology described in this manuscript were previously reported by the authors in another study [33].

### Clinical phenotyping

Clinical information was recorded at the time of sampling. The patients’ disease phenotype was determined guided by the leading symptoms categorised as proposed in the guidelines for the management of DADA2 [13]. The DADA2 activity index DADA2AI was determined by the treating physician and documented as described by Bucciol et al. [14].

### Sanger sequencing

Genomic DNA samples were prepared from heparinized peripheral blood following the instructions of the QIAamp DNA Blood Mini kit (#51104; QIAGEN, Hilden, Germany). Primers were designed with the help of Oligo Primer Analysis Software version 7 (Molecular Biology Insights, Colorado Springs, CO, USA). ADA2-specific gDNA amplification was performed using Platinum™ SuperFi™ PCR Master Mix (#12358010; Thermo Fisher Scientific, Waltham, MA, USA). PCR products were purified using the QIAquick PCR purification kit (#28106; QIAGEN). Sanger sequencing was performed on an ABI 3730 XL Genetic Analyser (Applied Biosystems, Waltham, MA, USA) at LGC Genomics (Berlin, Germany). Sequencing data were analysed using Chromas 2.6.5 (http://www.technelysium.com.au).

### Cell culture

Peripheral blood mononuclear cells (PBMCs) were isolated by density gradient centrifugation using Lymphoprep™ (#1114546; PROGEN, Heidelberg, Germany) in SepMate™ isolation tubes (#85450; STEMCELL Technologies, Vancouver, Canada) according to the manufacturer’s instructions. Prior to monocyte isolation, the final centrifugation step was performed at 300xg for 10 min at 4 °C. CD14+ monocytes were isolated magnetically by positive selection using human CD14 MicroBeads (#130-050-201; Miltenyi Biotec, Bergisch Gladbach, Germany) on LS columns (#130-042-401; Miltenyi Biotec) according to the manufacturer’s protocol. The cells were eluted into complete medium (RPMI 1640 medium (#61870044; Gibco, Carlsbad, CA, USA) supplemented with 10% foetal calf serum (FCS) (#S181BH-500, Biowest, Nuaillé, France) and 1% penicillin-streptomycin (#15140122; Gibco). Purity > 97% of CD14+ monocytes after magnetic sorting was verified by flow cytometry (anti-CD14-FITC clone MφP9, 1:40, #345784; BD Biosciences). For macrophage differentiation, 3 × 10^5^ CD14+ monocytes were seeded in a 12-well plate in 1 mL complete medium containing 20 ng/mL GM-CSF (#300-03; Peprotech, Rocky Hill, NJ, USA). The cells were differentiated for ten days, with medium changes every three days. U-937 cells and Jurkat cells (ATCC, Manassas, VA, USA) were cultured in complete RPMI medium.

### Transfection

The plasmid expressing myc-DDK-tagged wild-type ADA2 (transcript variant 3, NM_001282225) was purchased from OriGene Technologies (#RC238645). Pathogenic *ADA2* variants were created by site-directed mutagenesis using the Q5® Site-Directed Mutagenesis Kit (#E0554; New England Biolabs) according to the manufacturer’s instructions. Stable competent E. coli (#C3040H, New England Biolabs) were transformed with the generated constructs. Plasmid DNA was purified with the help of the QIAprep Spin Miniprep Kit (#27104, QIAGEN). Successful mutagenesis was verified by Sanger sequencing (LGC Genomics). HEK293T cells were seeded at 2.5 × 10^5^ cells/well in 2 mL in a 6-well plate 24 h prior to transfection. The cells were transfected with 1 µg plasmid DNA, using Lipofectamine™ 2000 Transfection Reagent (#11668019; Invitrogen) according to the manufacturer’s instructions. The medium was changed after 24 h. Supernatants were collected 72 h after transfection.

### Generation of ADA2^-/-^ cell lines by CRISPR/Cas9

Single-guide RNAs targeting *ADA2* from the human CRISPR Brunello library (#73179; addgene, Watertown, MA, USA) [34] were cloned into the lentiCRISPRv2 puro plasmid. lentiCRISPRv2 puro was a gift from Brett Stringer (plasmid #98290; addgene; http://n2t.net/addgene:98290; RRID:Addgene_98290). U-937 cells and Jurkat cells were transfected by electroporation using the Neon™ Transfection System (#MPK5000; Thermo Fisher Scientific) according to the manufacturer’s instructions [33].

### Transduction

Pathogenic *ADA2* variants were created by site-directed mutagenesis as described above. Fragments containing the sequence coding for the untagged ADA2 protein were then generated using the following primers and CloneAmp™ HiFi PCR Premix (#639298; Takara Bio):

ORF_ADA2_PSPXI_F_GGTGGTACTCGAGTATGTTGGTGGATGGCCCATCTGA; ORF_ADA2_PmeI_R_GGTGGTGTTTAAACCTTTGTAGCCACATCTGCTATGAACTT.

The fragments were cleaned up using the QIAquick PCR Purification Kit (#28104; QIAGEN) and cloned into the p3D lentiviral expression vector as follows: Restriction of the expression vector was performed in CutSmart buffer (#B7204; New England Biolabs) with PspX1 (#R0656; New England Biolabs) and PmeI (#R0560; New England Biolabs) at 37 °C overnight followed by 20 min heat inactivation at 65 °C. Dephosphorylation of 5´-ends was performed using rSAP (#M0371; New England Biolabs) at 37 °C overnight followed by 20 min heat inactivation at 65 °C. The PCR fragments were subsequently inserted into the expression vector using T4 ligase (#M0202; New England Biolabs) for 10 min at room temperature followed by 10 min heat inactivation at 65 °C. After DNA cleanup (#28104; QIAGEN), stable competent E. coli (#C3040H, New England Biolabs) were transformed with the generated plasmids. Plasmid DNA was purified with the help of the QIAprep Spin Miniprep Kit (#27104, QIAGEN). Sanger sequencing (LGC Genomics) was performed to confirm that the genetic information for *ADA2* was intact and that the respective mutations were present. Lentiviral particles were generated by transfecting HEK293T cells with the plasmids p3A (Gag-Pol), p3B (Rev), p3C (VSV-G) and p3D (expression vector) at a ratio of 1:1:1:3 using Lipofectamine™ 2000 Transfection Reagent (#11668019; Invitrogen). Supernatants containing lentiviral particles were harvested 48 and 72 h after transfection. The viral titre was determined using the qPCR Lentivirus Titer Kit (#LV900; Applied Biological Materials). Viral supernatants were prepared at MOI = 40 with 8 µg/mL polybrene and 10^6^ U-937 cells were transduced by spinoculation at 700xg for 2 h at 32 °C. After 4 h at 37 °C, the supernatant was replaced by fresh complete RPMI medium. Selection with 1 µg/mL puromycin (#ant-pr-1; InvivoGen) was started 48 hours after the transduction.

### Flow cytometry

Cells were placed on ice at the end of the incubation period and washed with PBS prior to staining with the following primary antibodies: anti-CD4-APC-H7 (clone RPA-T4, 1:20, #560158; BD Biosciences, Franklin Lakes, NJ, USA; RRID: AB_1645478) anti-CD8-PE-Cy5.5 (clone RPA-T8, 1:20, #35-0088-42; Thermo Fisher Scientific; RRID: AB_11218701), anti-CD14-FITC (clone MφP9, 1:40, #345784; BD Biosciences), anti-CD19-BV421 (clone HIB19, 1:20, #302234; BioLegend, San Diego, CA, USA; RRID: AB_11142678). The viability staining was performed with the following reagents: Zombie Violet™ Fixable Viability Dye (1:1000, #423113, BioLegend) and Annexin V-APC (1:100, #550474, BD Biosciences; RRID: AB_2868885) in 1X Annexin V Binding Buffer (#556454, BD Biosciences). Prior to flow cytometric analysis, the cells were fixed in 1% formaldehyde in Annexin V Binding Buffer.

ROS content was determined with the help of the Total ROS Assay Kit 520 nm (#88-5930-74, Thermo Fisher Scientific) used according to the manufacturer’s instructions.

The measurements were performed on Canto II and Symphony flow cytometers (BD Biosciences). The data were analysed with FlowJo software (version 10.8.1). For analysis, debris was excluded by forward and sideward scatter and singlets were identified before determining the percentage of dead cells.

### Cell proliferation assay

Cell proliferation was assessed by CellTrace™ Yellow Cell Proliferation Assay (#C34573; Thermo Fisher Scientific). U-937 cells were stained in CellTrace working solution at 1:500 in PBS according to the manufacturer’s instructions. Samples were taken after 10 minutes (baseline) and 2, 4, and 6 days respectively. Cells were prepared for flow cytometry by additionally staining with a fixable viability dye (see above) and fixing in 2% formaldehyde in PBS.

### Cell death assay

Cells were seeded in a 24-well plate at 2.5 × 10^5^ cells in 500 µL complete medium. Prior to induction of cell death, the cells were incubated for 30 min with inhibitors of different forms of RCD as indicated: 20 µM Z-VAD-FMK (#S7023; Selleck Chemicals GmbH, Houston, TX, USA), 20 µM Z-DEVD-FMK (S7312; Selleck Chemicals GmbH), 10 µM necrostatin-1s (#S8641; Selleck Chemicals GmbH), 1 µM necrosulfonamide (#S8251; Selleck Chemicals GmbH), 20 µM Z-YVAD-FMK (#S8507; Selleck Chemicals GmbH), 50 µM deferoxamine (#S5742; Selleck Chemicals GmbH), 2 µM ferrostatin-1 (#S7243; Selleck Chemicals GmbH).

Apoptosis was induced by 30-minute incubation with 100 nM birinapant (#S7015; Selleck Chemicals GmbH) followed by 4 h incubation with 20 ng/mL TNF-α (#rcyc-htnfa; InvivoGen, San Diego, CA, USA). Necroptosis was induced by 30 min incubation with 100 nM birinapant (#S7015; Selleck Chemicals GmbH) and 20 µM Z-VAD-FMK (#S7023; Selleck Chemicals GmbH) followed by 4 h incubation with 20 ng/mL TNF-α (#rcyc-htnfa; InvivoGen).

TNF inhibition was achieved by incubation with 5 μg/mL adalimumab (#A2010, Selleck Chemicals GmbH). Ruxolitinib (5 μM, #S1378, Selleck Chemicals GmbH) was used to block JAK1/2 signalling and the STING-inhibitor H-151 (1 μM, #inh-h151, InvivoGen) served to examine DNA sensing. Effective concentrations were determined by titration after stimulation of the respective pathways (Supplementary Figure S2A+B and S3).

### Immunoblotting

Whole cell lysates were obtained by lysing 1×10^6^ cells in 25 µL RIPA buffer (150 mM NaCl, 1% Triton X-100, 0.5% sodium deoxycholate, 0.1% SDS, pH 8.0) containing protease inhibitor (#78429; Thermo Fisher Scientific) for 30 min on ice, followed by centrifugation at 13,500xg for 20 min at 4 °C. Bolt™ LDS sample buffer (#B0007; Thermo Fisher Scientific) mixed with Bolt™ Sample Reducing Agent (#B0009; Thermo Fisher Scientific) was added to the samples prior to gel electrophoresis. 1200 µL ice-cold acetone were added to 300 µL urine and the samples were inverted and vortexed thoroughly for protein precipitation. After overnight incubation at -20 °C, the samples were spun for 15 min at 15,000xg at 4 °C. Supernatants were discarded, pellets resuspended in 75 µL 1X sample buffer and placed at 95 °C for 10 min. 20 µL were loaded onto the gel. SeeBlue™ Plus2 Pre-stained Protein Standard (#LC5925; Thermo Fisher Scientific) was used as protein molecular weight marker. Proteins were transferred onto PVDF transfer membranes. Ponceau S was used according to the manufacturer’s instructions. The membranes were probed with the following primary antibodies: anti-ADA2 (clone EPR25430-131, #ab288296, 1:1000; abcam, Cambridge, UK), anti-pMLKL (S358) (clone: D6H3V, #91689, 1:1000; Cell Signaling Technology, Danvers, MA, USA; RRID:AB_2732034), anti-MLKL (clone: D2I6N, #14993, 1:1000; Cell Signaling Technology; RRID:AB_2721822), anti-pRIP (Ser166) (clone: D1L3S, #65746, 1:1000; Cell Signaling Technology; RRID:AB_2799693), anti-RIP (clone: D94C12, #3493, 1:1000; Cell Signaling Technology; RRID:AB_2305314), anti-cleaved caspase-3 (Asp175) (clone: 5A1E, #9664, 1:1000; Cell Signaling Technology; RRID:AB_2070042), anti-caspase-3 (#9662, 1:1000; Cell Signaling Technology; RRID:AB_331439), anti-cyclophilin A (#ab41684, 1:1000; abcam; RRID:AB_879768), anti-GAPDH (clone: 7B, #sc-69778, 1:500; Santa Cruz Biotechnology, Dallas, TX, USA; RRID:AB_1124759), and anti-β-actin (clone: AC-15, #A5441, 1:9,000; Sigma-Aldrich, St. Louis, MO, USA; RRID: AB_476744) at 4 °C overnight or at room temperature for two hours. The membranes were washed and incubated with the respective HRP-coupled secondary antibodies for one hour at room temperature: goat anti-rabbit IgG H&L (#ab205718, 1:5000; abcam; RRID: AB_2819160) or Goat Anti-Mouse IgG (H + L) (#71045, 1:5000; Sigma-Aldrich; RRID: AB_11211441). Protein expression was visualised by enzymatic chemiluminescence using Pierce^TM^ ECL western blotting substrate (#32106; Thermo Fisher Scientific) or SuperSignal™ West Pico PLUS Chemiluminescent Substrate (#34580; Thermo Fisher Scientific) in a ChemiDoc XRS+ Imaging System (Bio-Rad). Protein bands were quantified with the help of Image J. The full length uncropped western blots are provided in the Supplementary Material.

### Microscopy

U-937 cells were cultured in complete RPMI medium. The immunofluorescence staining was performed in V-shaped 96-well plates in the dark at room temperature (RT). Cells were harvested, fixed in 4% (v/v) paraformaldehyde (Electron Microscopy Sciences) for 10 min, and permeabilized with PBS containing 0.1% Tween®20 (Qbiogene Inc.) for 10 min. To prevent nonspecific antibody binding, cells were incubated in blocking buffer (1x PBS supplemented with 5% FCS and 5 mg/mL human IgG; IgG1 66.6%, IgG2 28.5%, IgG3 2.7%, IgG4 2.2%; Grifols) for 30 min. Nuclear staining was performed using DAPI (1 µg/mL in PBS / 0.1% Tween®20) for 15 min. For imaging, stained cells were transferred onto glass slides using a Cytospin 4 centrifuge (Thermo Fisher Scientific) at 800 rpm for 3 min with medium acceleration. Images were acquired with a laser scanning confocal fluorescence microscope (LSM 710, Carl Zeiss).

In addition, cell morphology was evaluated in the culture plate by brightfield microscopy on an EVOS M7000 Microscope Imaging System (#AMF7000; Invitrogen, Waltham, MA, USA) using the 20x objective.

### qPCR

5–10×10^5^ cells were lysed in TRIzol™ Reagent (#15596018; Thermo Fisher Scientific) for 3 min at room temperature and homogenised before storage at -80 °C. RNA was extracted using the PureLink™ RNA Mini Kit (#12183018 A; Thermo Fisher Scientific) according to the manufacturer’s instructions. cDNA was generated from 20 ng RNA using the SuperScript™ VILO™ cDNA Synthesis Kit (#11754050; Thermo Fisher Scientific). Quantitative polymerase chain reaction (qPCR) analysis was performed with SsoAdvanced™ Universal SYBR® Green Supermix (#1725271; Bio-Rad Laboratories, Hercules, CA, USA) and the following primers:

- IFI27_F_TCGCCTCGTCCTCCATAGCAG;
- IFI27_R_ AGTAGAACCTCGCAATGACAGCC;
- IFI44L_F_ATCTTAAAAGGTTGTATGCCAGA;
- IFI44L_R_ACTTGCTTCACTTTTGCCAA; IFIT1_F_ATGAGTACAAATGGTGATGA;
- IFIT1_R_AATTCAATCTGATCCAAGAC; ISG15_F_GGTGGACAAATGCGACGAACCTC;
- ISG15_R_CACACCCTCCAGCCCGCTCA; RSAD2_F_ GCGTCAACTATCACTTCACTCG;
- RSAD2_R_ CAGGTATTCTCCCCGGTCT; SIGLEC1_F_ TCTTGCCCAAGCTTCTCCTC;
- SIGLEC1_R_GTAGTACCAGATGGCCGTGA;
- GAPDH_F_GTCTCCTCTGACTTCAACAGCG;
- GAPDH_R_ACCACCCTGTTGCTGTAGCCAA

For all conditions, three technical replicates were measured. The experiment was run on a QuantStudio™ 3 Real-Time PCR System (Thermo Fisher Scientific) and analysed using the QuantStudio™ Design & Analysis Software v1.5.2. The relative abundance of the respective gene was normalised to the expression level of *HPRT1* or *GAPDH*. and different conditions were compared using the 2^-ΔΔCt^ method [35].

For analysis of whole blood samples, total RNA was extracted from PAXgene RNA tubes using the PAXgene Blood RNA Kit, v2 (PreAnalytiX, Qiagen/ BD). The median fold change of the six interferon-stimulated genes *IFI27*, *IFI44L*, *IFIT1*, *ISG15*, *RSAD2*, *SIGLEC1* when compared to expression in healthy control samples was used to create an interferon score for each individual [36].

### Metabolomics

Magnetically sorted CD14+ monocytes from healthy control and DADA2 patients were seeded at 3 × 10^5^ cell/well in 2 mL complete RPMI medium in a 6-well plate and cultured for 10 days with 20 ng/mL GM-CSF. Medium changes were performed every three days. On day 10, the medium was replaced by RPMI medium containing ^13^C-labelled glucose supplemented with 10% dialysed medium (three replicates). One well was incubated with RPMI medium containing ^12^C glucose at the same concentration. After 24 h incubation, the medium was removed, and the cells washed with ice-cold 0.9% NaCl solution. The cells were incubated in 300 µL ice-cold cellular extraction buffer (80% methanol containing 2 µM d27 myristic acid). The extraction mix was then centrifuged at 20.000xg for 15 min at 4 °C and the supernatants stored for metabolomics analysis (see below). The pellet was lysed in 200 mM NaOH solution and BCA assay performed for protein quantification.

U-937 cells were seeded at 1.5 × 10^6^ cells/well in ^13^C or ^12^C glucose-containing medium. After 24 h incubation the cells were washed in ice-cold PBS and centrifuged at 1500xg for 5 minutes. 150 µL extraction buffer (50 : 30 : 20 methanol : acetonitrile : water, containing 10 mM Tris-HCl pH 9.4) were added and the samples vortexed thoroughly and stored overnight at -80 °C. The samples were then centrifuged at >12.000xg for 15 min at 4 °C and the supernatants used for metabolomics analysis (see below). The protein pellet was used for the BCA assay as above.

10 μL of each sample were loaded into a Dionex UltiMate 3000 LC System (Thermo Fisher Scientific) equipped with a C-18 column (Acquity UPLC -HSS T3 1. 8 μm; 2.1 × 150 mm, Waters) coupled to a Q Exactive Orbitrap mass spectrometer (Thermo Scientific) operating in negative ion mode. A step gradient was carried out using solvent A (10 mM TBA and 15 mM acetic acid) and solvent B (100% methanol). The gradient started with 5% of solvent B and 95% solvent A and remained at 5% B until 2 min post injection. A linear gradient to 37% B was carried out until 7 min and increased to 41% until 14 min. Between 14 and 26 minutes the gradient increased to 95% of B and remained at 95% B for 4 min. At 30 min the gradient returned to 5% B. The chromatography was stopped at 40 min. The flow was kept constant at 0.25 mL/min and the column was placed at 40 °C throughout the analysis. The MS operated in full scan mode (m/z range: [70.0000-1050.0000]) using a spray voltage of 4.80 kV,capillary temperature of 300 °C, sheath gas at 40.0, auxiliary gas at 10.0. The AGC target was set at 3.0E + 006 using a resolution of 140000, with a maximum IT fill time of 512 ms. Data collection was performed using the Xcalibur software (Thermo Fisher Scientific). The data analyses were performed by integrating the peak areas (El-Maven – Polly - Elucidata).

### Statistical analysis

Statistical analysis was performed in R and GraphPad Prism. Normality testing was performed by Q-Q-plot. Where a normal distribution could be assumed, samples were compared by t-test. Otherwise, Wilcoxon signed rank test was used for differential analysis of paired samples and Mann-Whitney-U test for differential analysis of unpaired samples.

For the tracer metabolomics experiments, the raw MS data was used as input to IsoCor v2 to perform natural abundance correction [37]. The following parameters were used: resolution reference: 140000, mz reference: 200, isotopic purity of the tracer: 100%. For each metabolite and each cell line, a t-test was performed to determine differences in mean enrichment between HC and patients.

Single-cell RNA sequencing data was provided by Watanabe et al. in the form of the output of the cellranger pipeline [11]. scAR was used for ambient RNA removal and scDblFinder to identify and remove doublets (default parameters) [38, 39]. Further quality control was performed according to the recommendations of Heumos et al. [40]. DEA was carried out by first creating pseudobulks for each donor, and then using the limma-voom method with the empirical Bayes procedure [41, 42]

## Supplementary information

Supplemental material

Uncropped blots

## Data Availability

The authors provide a supplementary file containing the original uncropped blots of all the presented data. Further information is available upon request.
