# Supplementary material for: ADA2-deficient cells exhibit increased levels of cell death and metabolic disturbances
Source: Cell Death Discov. 2026 Mar 23;12:167. doi: 10.1038/s41420-026-03027-9 (PMC13039166; doi:10.1038/s41420-026-03027-9)
Supplement: Supplementary file 1 — Supplemental material [file 41420_2026_3027_MOESM1_ESM.pdf]

Supplementary material

This file contains Supplementary Figures S1-S7 and Supplementary Table S1.

Supplementary Figures

Supplementary Figure S1

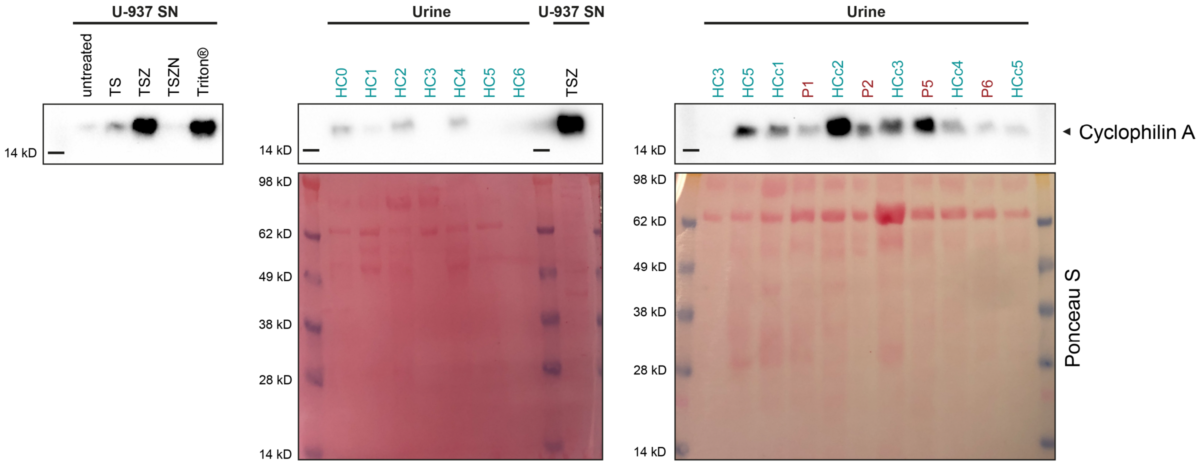

Supplementary Figure S1: Urine levels of cyclophilin A as a marker of necroptosis. Western blot analysis of cyclophilin A in urine from healthy controls (HC) and DADA2 patients (P). Ponceau S staining was performed on the membrane for normalization of protein levels. As a positive control, the left panel shows cyclophilin A levels in the supernatant of U-937 cells after induction of apoptosis (TS) and necroptosis (TSZ) by stimulation with 100 nM birinapant (SM) ± 20 μM Z-VAD-FMK followed by 20 ng/mL TNF-α. Inhibition of necroptosis was achieved by adding 10 μM necrostatin-1s (TSZN). Cell permeabilization with Triton® X-100 was used as an internal control.

Supplementary Figure S2

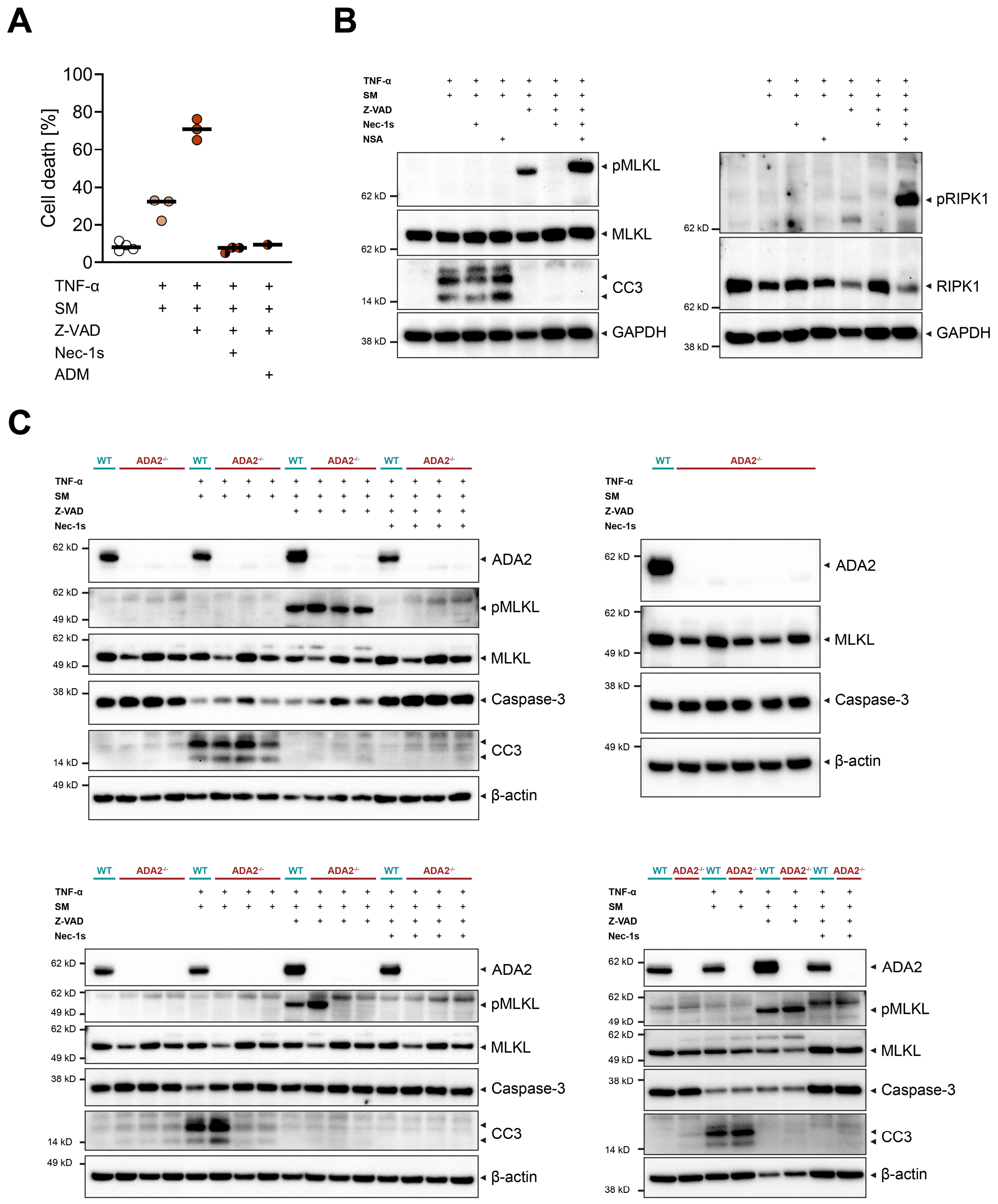

Supplementary Figure S2: Induction of necroptosis *in vitro* in U-937 cells. (A) Cell death of ADA2$^{WT/WT}$ U-937 cells after induction of necroptosis by 30-minute incubation with 100 nM birinapant (SM) and/or 20 µM Z-VAD-FMK (Z-VAD) followed by 4-hour incubation with 20 ng/mL TNF-α. 10 µM Necrostatin-1s (Nec-1s) or 5 µg/mL adalimumab (ADM) were added 30 minutes before where applicable. Dead cells were identified as Annexin V / Zombie double-

positive by flow cytometry. Median is shown. (B) Induction of necroptosis in $ADA2^{WT/WT}$ U-937 cells by western blot. Conditions as in A. Necrosulfonamide (NSA) was used at 1 µM. (C) Induction of necroptosis in $ADA2^{WT/WT}$ and $ADA2^{-/-}$ U-937 cells by western blot. Conditions as in A. The blots displayed here underlie the plots shown in **Figure 3B**.

Legend: CC3, cleaved caspase-3; MLKL, mixed lineage kinase domain like pseudokinase; pMLKL, phosphorylated mixed lineage kinase domain like pseudokinase; pRIPK1, phosphorylated receptor-interacting serine/threonine-protein kinase 1; RIPK1, receptor-interacting serine/threonine-protein kinase 1.

Supplementary Figure S3

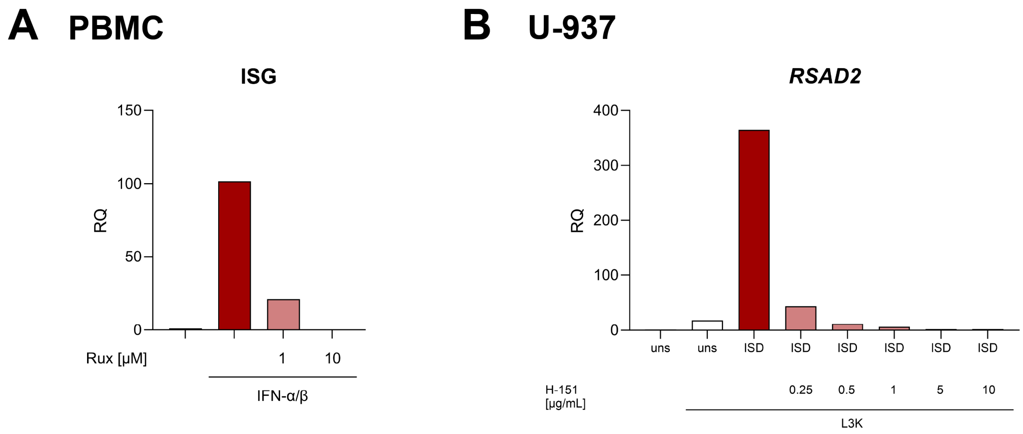

Supplementary Figure S3: Inhibition of type I interferon signalling by ruxolitinib and H-151. (A) Healthy control PBMCs were cultured with or without 5.000 U/mL interferon(IFN)-alpha and 5.000 U/mL interferon-beta as well as 1 µM or 10 µM ruxolitinib (Rux) for 24 hours. Expression of interferon stimulated genes (ISG) was determined by qPCR. mRNA expression was normalized to *HPRT1* and is depicted relative to the untreated control (RQ). ISG represents the mean gene expression of *IFI27*, *IFI44L*, *IFIT1*, *ISG15*, *RSAD2* and *SIGLEC1*. (B) U-937 cells were stimulated with 1 µg interferon stimulatory DNA (ISD) introduced by lipofection and incubated with increasing concentrations of H-151 over 24 hours. The graph shows *RSAD2* gene expression determined by qPCR. mRNA expression was normalized to *HPRT1* and is depicted relative to the untreated control (RQ).

Supplementary Figure S4

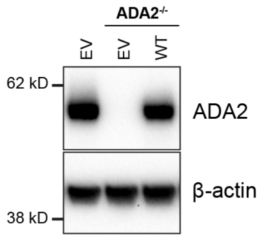

Supplementary Figure S4: Transduction of ADA2$^{-/-}$ U-937 cells with WT *ADA2*. Western blot showing ADA2 protein expression in whole cell lysates of ADA2$^{WT/WT}$ U-937 cells and ADA2$^{-/-}$ U-937 cells transduced with empty vector (EV) and wild-type *ADA2* (WT), respectively.

Supplementary Figure S5

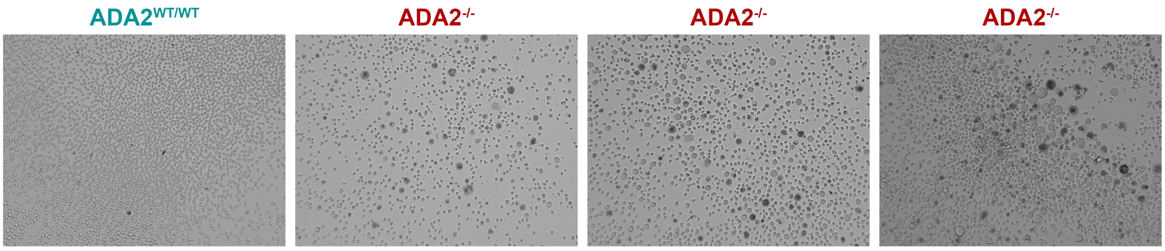

Supplementary Figure S5: Morphology of ADA2$^{-/-}$ U-937 cells. Microscopy of ADA2$^{WT/WT}$ and ADA2$^{-/-}$ U-937 cells. Three different knock-out clones are shown. Imaging was performed under standard cell culture conditions.

Supplementary Figure S6

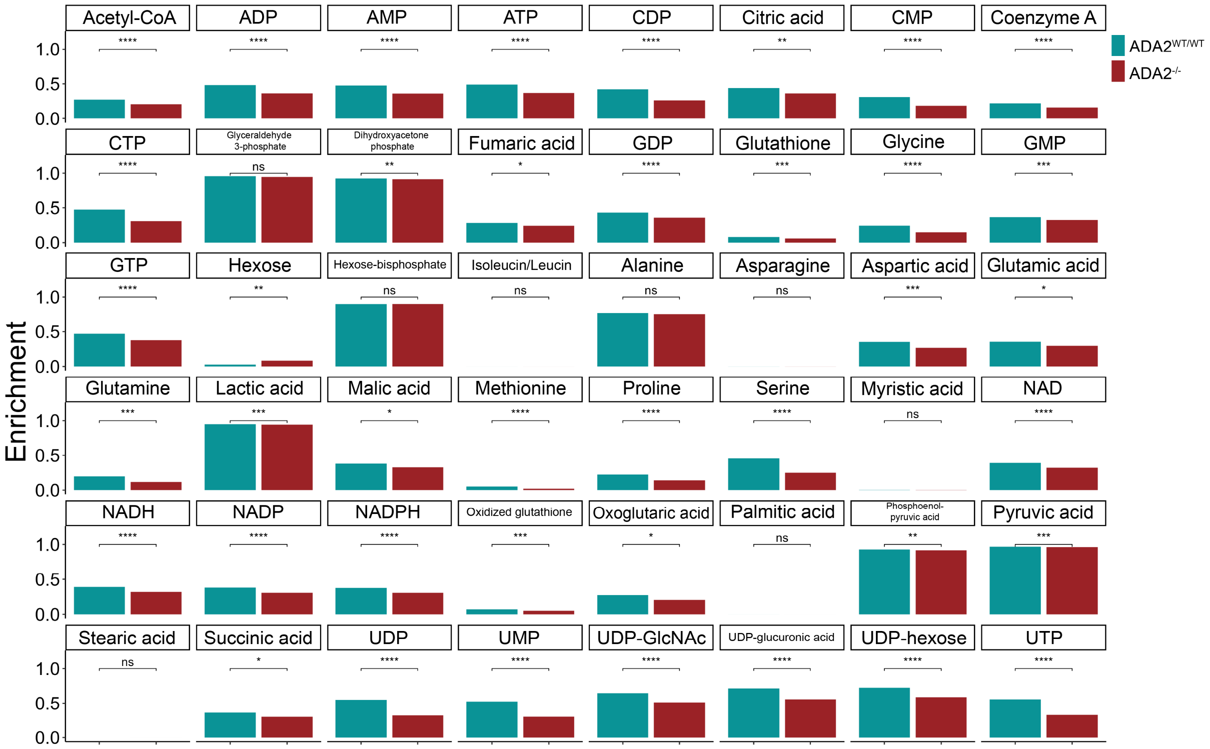

Supplementary Figure S6: Metabolite enrichment by tracer metabolomics of U-937 cells. Bar graphs depict mean enrichment of each metabolite, i.e. the mean molecular content in isotopic tracer in the metabolite. The analysis was performed in three technical replicates of ADA2$^{WT/WT}$ (n=1) and ADA2$^{-/-}$ (n=3) U-937 cells. Student's t-Test, *$p < 0.05$, **$p < 0.01$, ***$p < 0.001$, ****$p < 0.0001$.

Legend: UDP-GlcNAc, Uridine diphosphate-N-acetylglucosamine; UDP-glucuronic acid, Uridine disphosphate glucuronic acid; UDP-hexose, Uridine diphosphate hexose.

Supplementary Figure S7

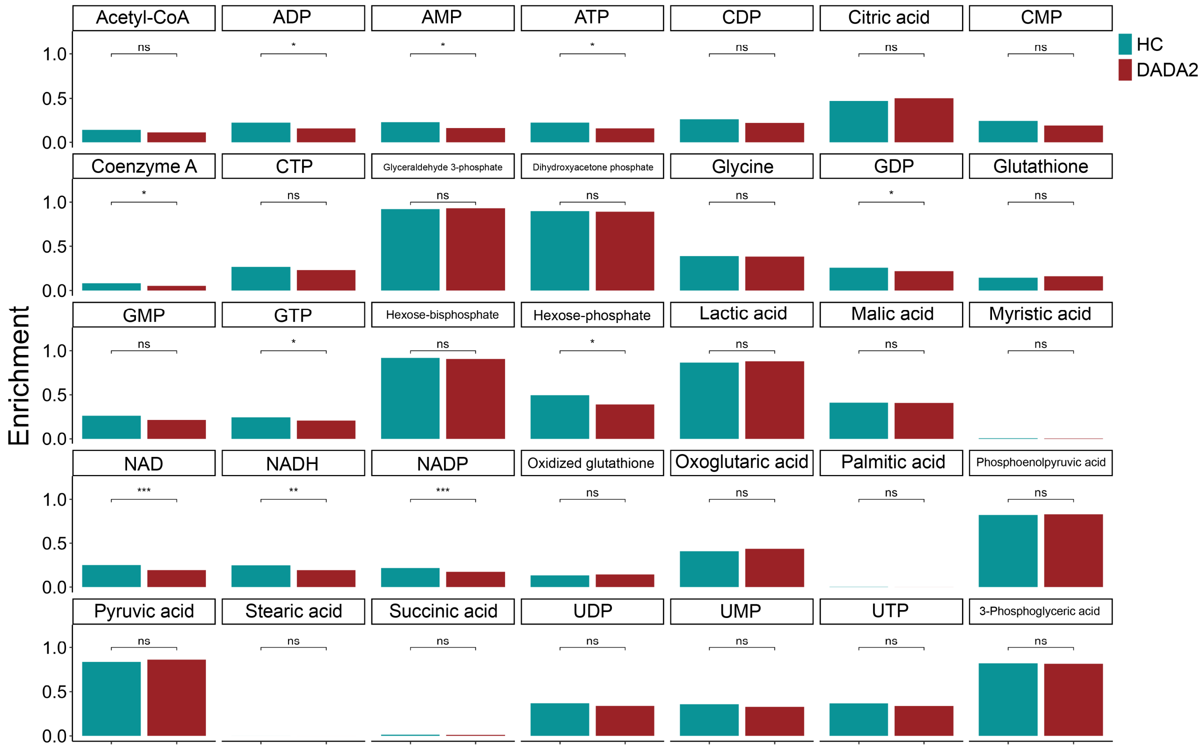

Supplementary Figure S7: Metabolite enrichment by tracer metabolomics of human monocyte-derived macrophages. Bar graphs depict mean enrichment of each metabolite, i.e. the mean molecular content in isotopic tracer in the metabolite. The analysis was performed in three technical replicates of healthy control (HC) (n=1) and DADA2 (n=1) monocyte-derived macrophages. Student's t-Test, $*p < 0.05$, $**p < 0.01$, $***p < 0.001$.

Supplementary Tables

Supplementary Table S1

ID	Age [years]	Sex	Genotype	Predominant phenotype
P1	12	M	c.973-2A>G/c.del1240-1442<br>splice site (intron 6)/del exon 9	Vasculitis, hepatosplenomegaly, hypogammaglobulinemia
P2	14	F	c.973-2A>G/c.del1240-1442<br>splice site (intron 6)/del exon 9	Vasculitis, warts, hypogammaglobulinemia, arthralgia
P3	26	M	c.140G>T/c.del1240-1442<br>p.G47V/del exon 9	Bone marrow failure, hypogammaglobulinemia, hepatosplenomegaly

P4	26	M	c.140G>T/c.140G>T<br>p.G47V/p.G47V	Hypogammaglobulinemia, hepatosplenomegaly
P5	6	F	c.140G>T/c.506G>A<br>p.G47V/p.R169Q	Hypogammaglobulinemia, hepatosplenomegaly, stroke
P6	9	F	c.140G>T/c.506G>A<br>p.G47V/p.R169Q	Bone marrow failure, hypogammaglobulinemia, hepatosplenomegaly
P7	32	F	c.973-2A>G/c.506G>A<br>splice site (intron 6)/p.R169Q	Stroke, hypertension
P8	16	M	c.139G>A/c.139G>A<br>p.G47R/p.G47R	Stroke, vasculitis
P9	10	F	c.973-2A>G/c.973-2A>G<br>splice site (intron 6)	Vasculitis, hypogammaglobulinemia
P10	NA	F	c.973-2A>G/c.506G>A<br>splice site (intron 6)/p.R169Q	Vasculitis, neutropenia

Part of this cohort was also reported in another study conducted by this research group.[1]

Supplementary References

1. Ehlers L, Hombrouck A, Wouters M, Pillay B, Delafontaine S, Bucciol G, et al. Human ADA2 deficiency is characterized by the absence of an intracellular hypoglycosylated form of adenosine deaminase 2 [Internet]. bioRxiv; 2024 [cited 2024 Oct 23]. p. 2023.10.25.564037. Available from: https://www.biorxiv.org/content/10.1101/2023.10.25.564037v3
