# Supplementary material for: ADA2-deficient cells exhibit increased levels of cell death and metabolic disturbances
Source: Cell Death Discov. 2026 Mar 23;12:167. doi: 10.1038/s41420-026-03027-9 (PMC13039166; doi:10.1038/s41420-026-03027-9)

Figure 3B

anti-pMLKL (S358) [D6H3V] + goat anti-rabbit (ab205718)

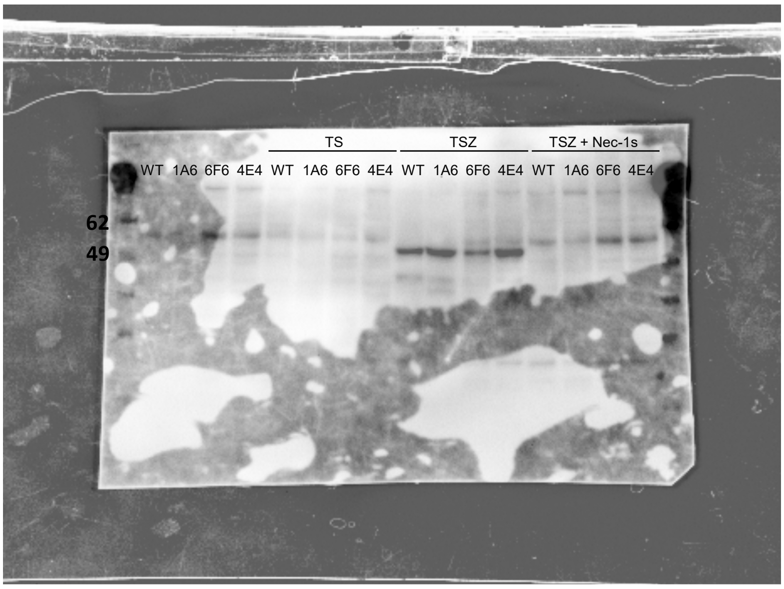

anti-MLKL [D2I6N] + goat anti-rabbit (ab205718)

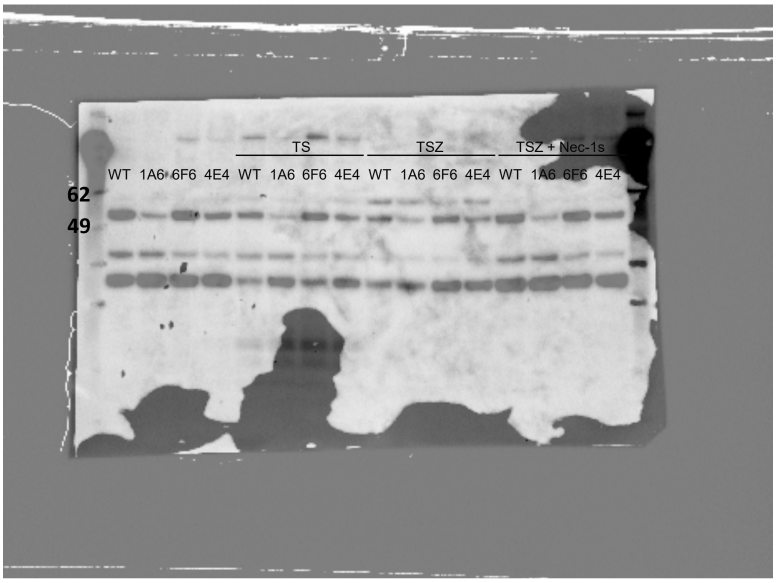

anti-cleaved caspase 3 (Asp175) [5A1E]
+ goat anti-rabbit (ab205718)

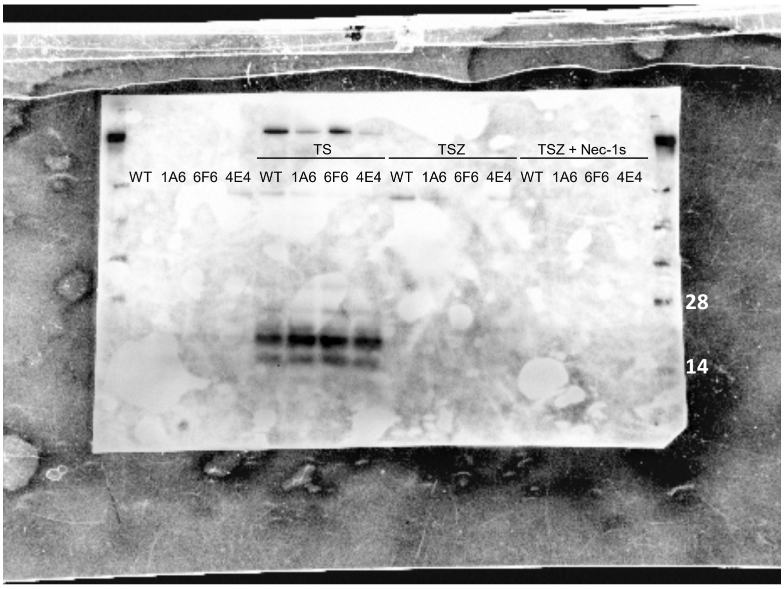

anti-caspase-3 (#9662) + goat anti-rabbit (ab205718)

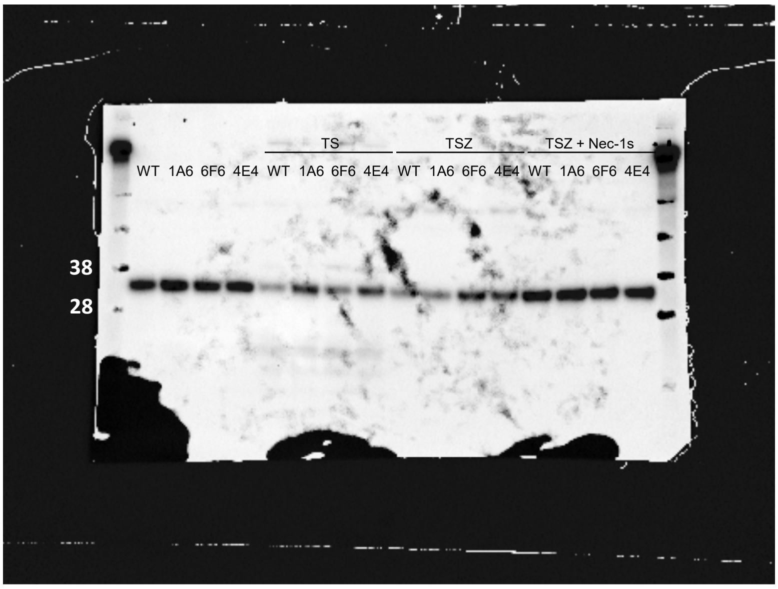

anti-ADA2 (ab288296) + goat anti-rabbit (ab205718)

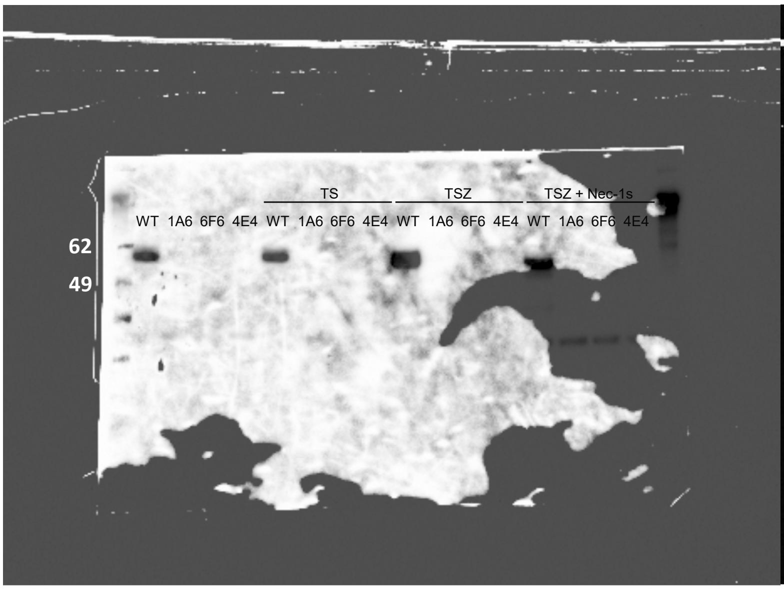

anti-β-actin (AC-15) + goat anti-mouse (#71045)

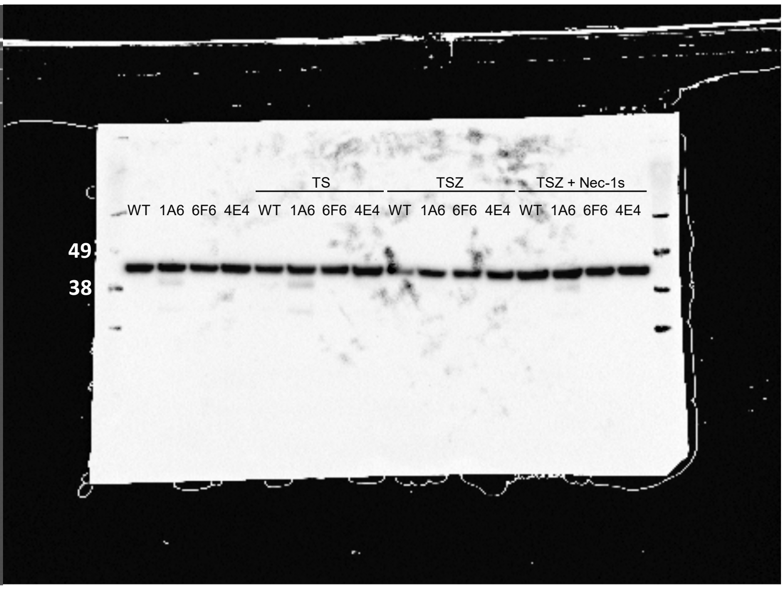

Figure S1

anti-cyclophilin A (ab41684) + goat anti-rabbit (ab205718)

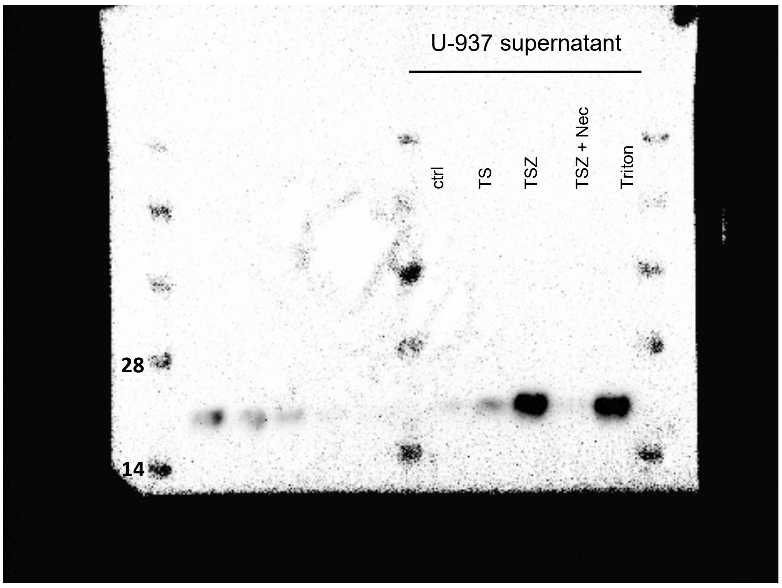

anti-cyclophilin A (ab41684) + goat anti-rabbit (ab205718)

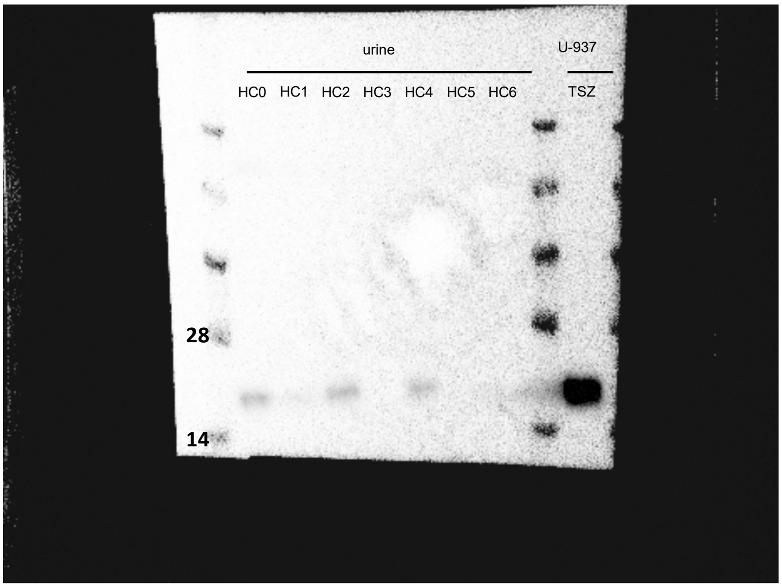

anti-cyclophilin A (ab41684) + goat anti-rabbit (ab205718)

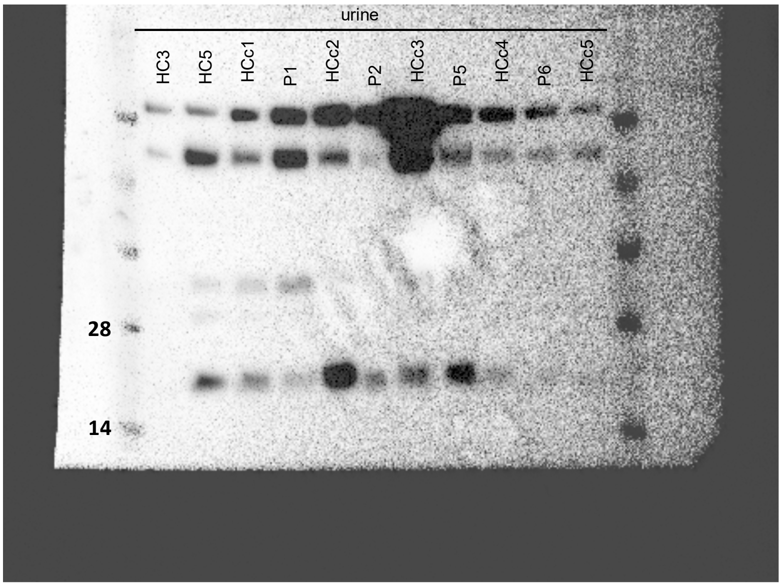

Figure S2B

anti-pMLKL (S358) [D6H3V] + goat anti-rabbit (ab205718)

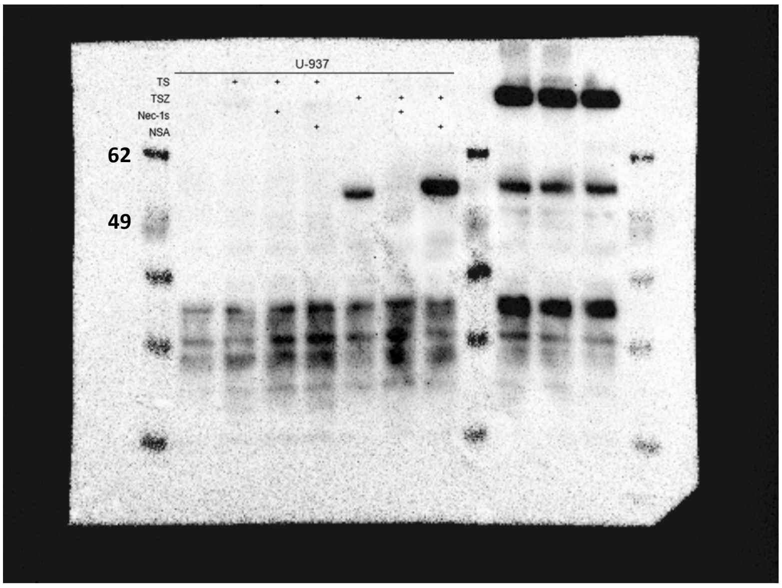

anti-MLKL [D2I6N] + goat anti-rabbit (ab205718)

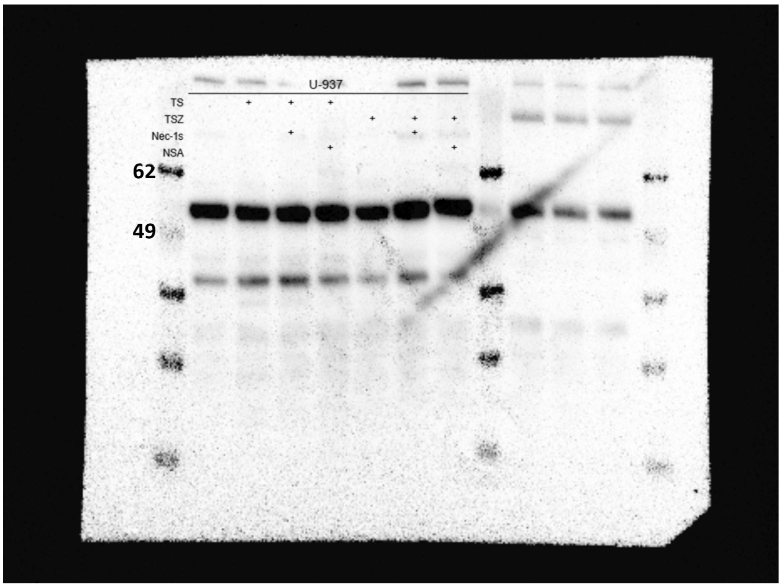

anti-cleaved caspase 3 (Asp175) [5A1E]
+ goat anti-rabbit (ab205718)

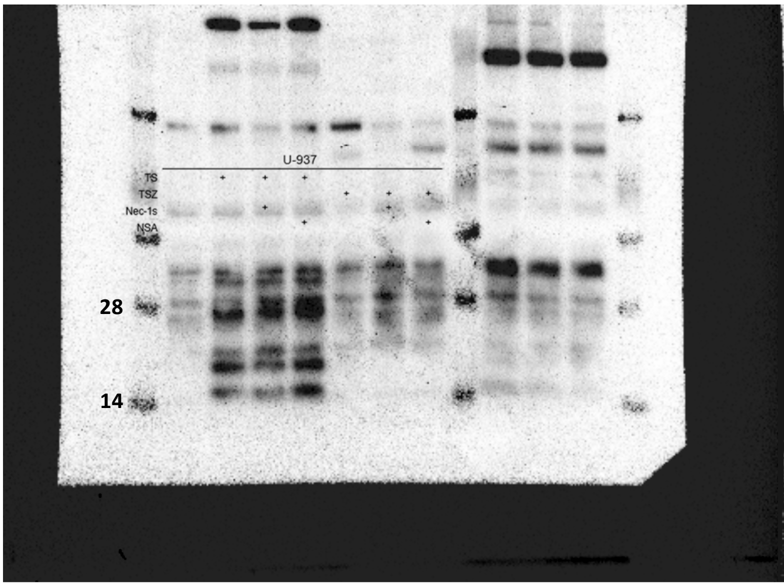

anti-GAPDH (7B) + goat anti-mouse (#71045)

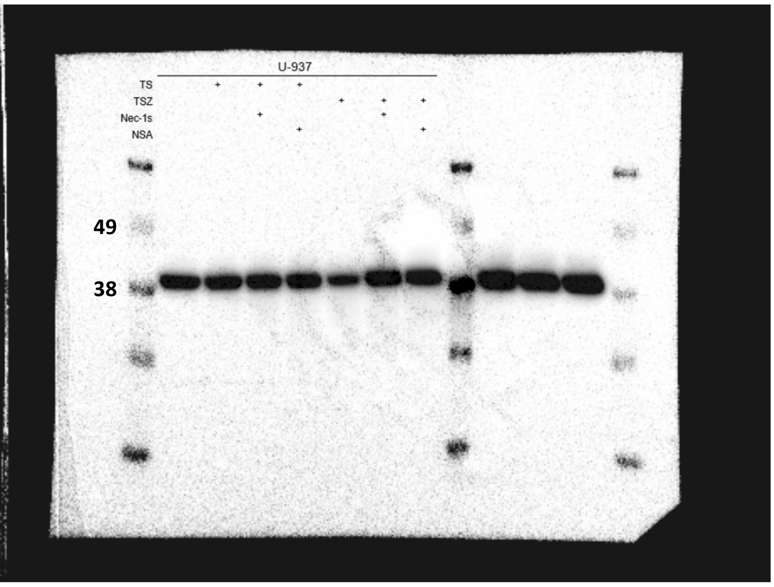

Figure S2B

anti-pRIP (Ser166) [D1L3S] + goat anti-rabbit (ab205718)

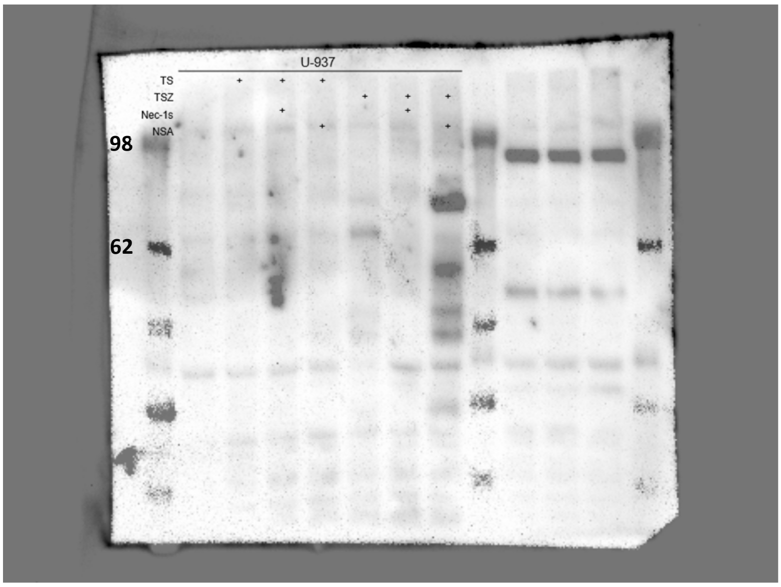

anti-RIP [D94C12] + goat anti-rabbit (ab205718)

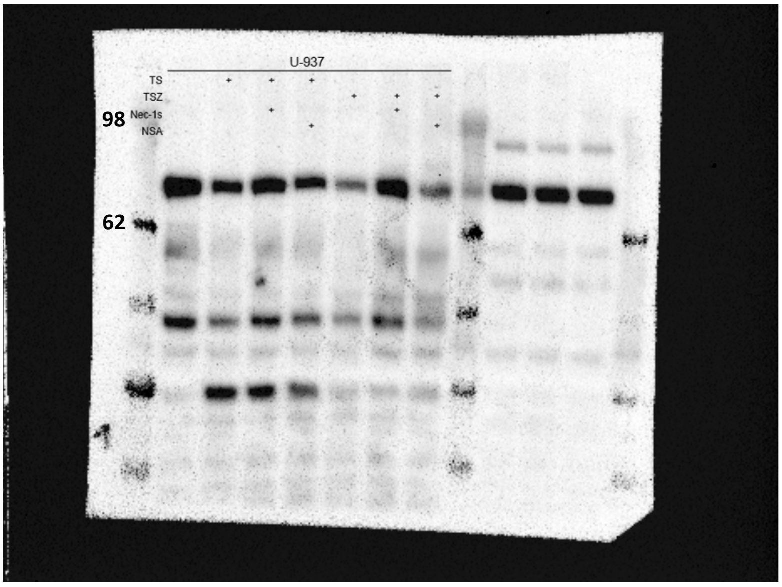

anti-GAPDH (7B) + goat anti-mouse (#71045)

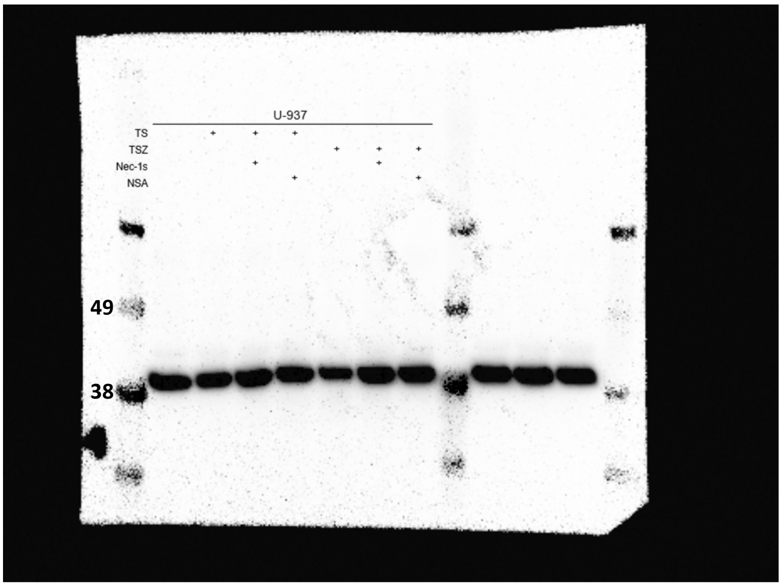

Figure S2C

anti-pMLKL (S358) [D6H3V] + goat anti-rabbit (ab205718)

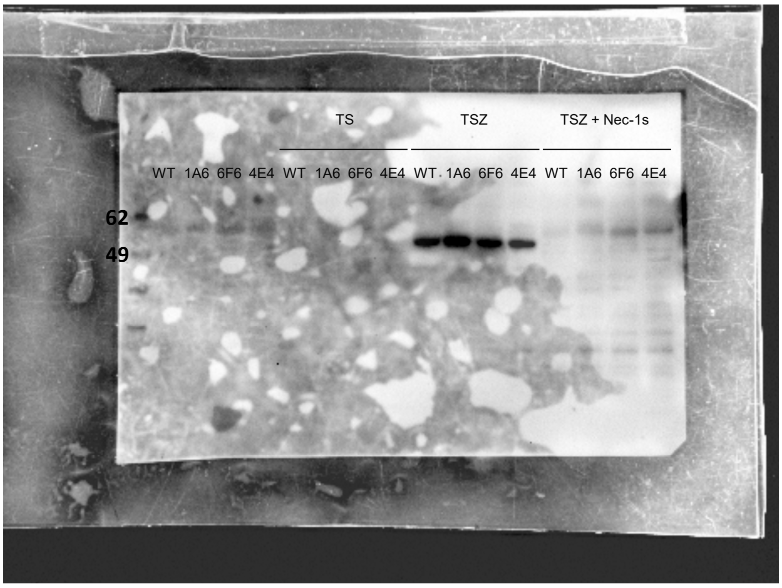

anti-MLKL [D2I6N] + goat anti-rabbit (ab205718)

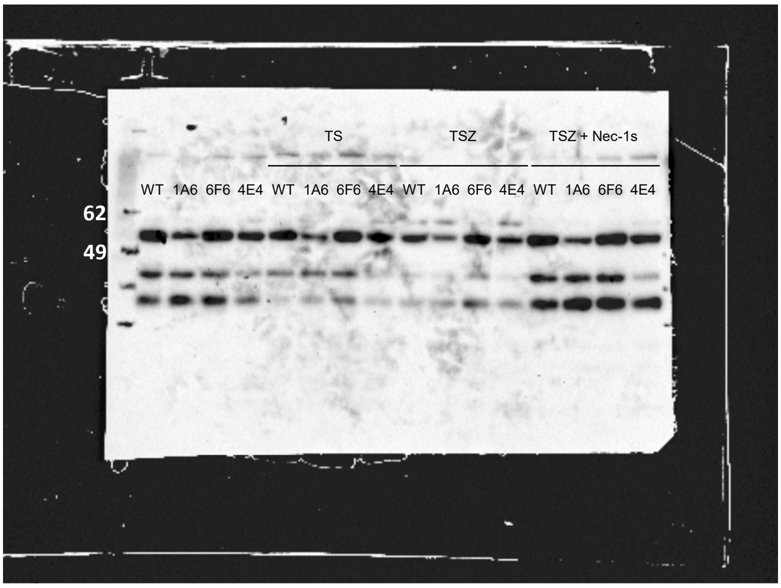

anti-cleaved caspase 3 (Asp175) [5A1E]
+ goat anti-rabbit (ab205718)

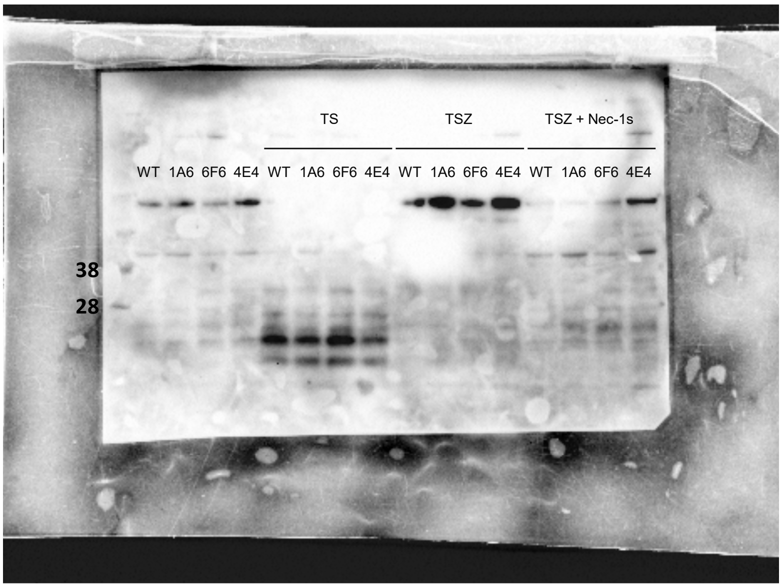

anti-caspase-3 (#9662) + goat anti-rabbit (ab205718)

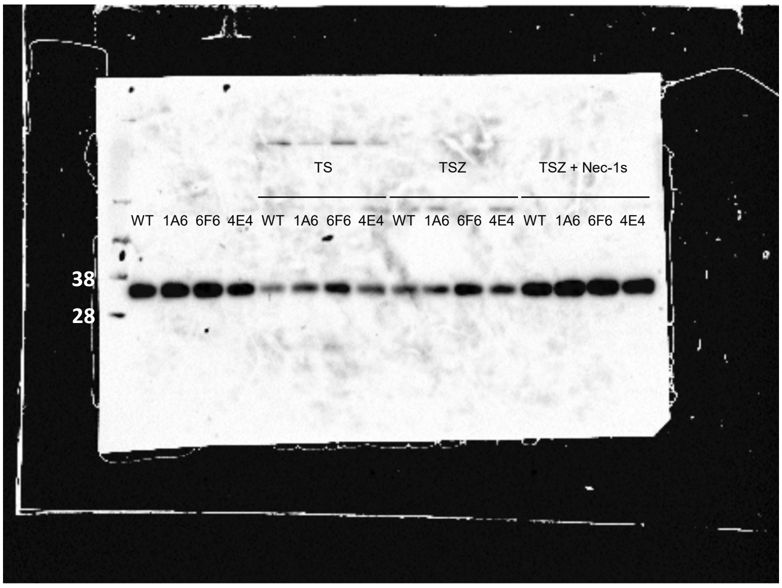

anti-ADA2 (ab288296) + goat anti-rabbit (ab205718)

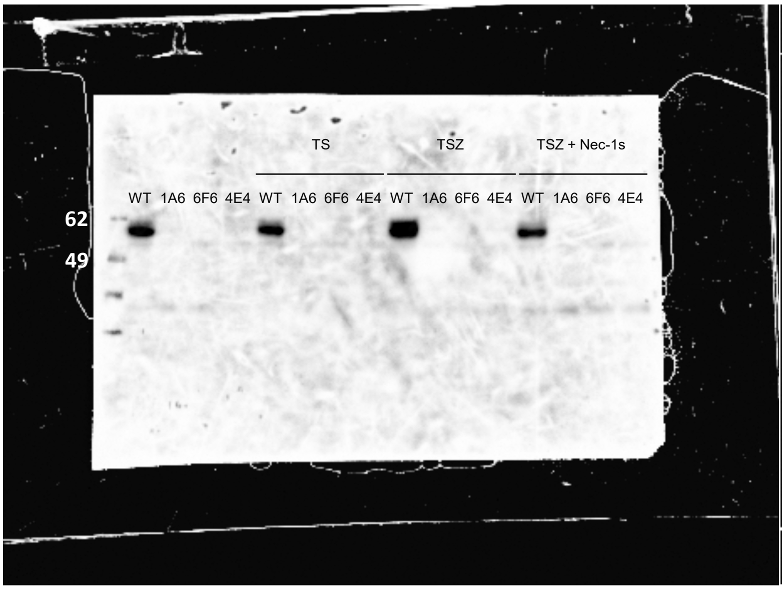

anti-β-actin (AC-15) + goat anti-mouse (#71045)

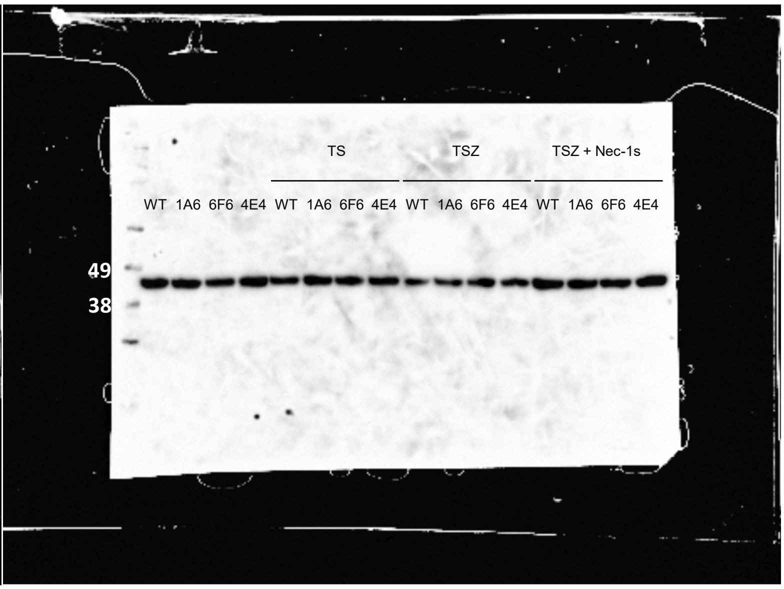

Figure S2C

anti-pMLKL (S358) [D6H3V] + goat anti-rabbit (ab205718)

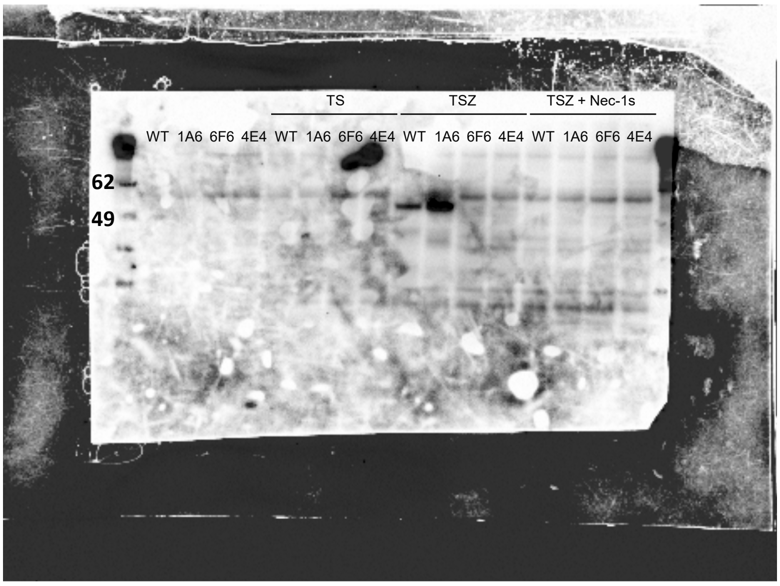

anti-MLKL [D2I6N] + goat anti-rabbit (ab205718)

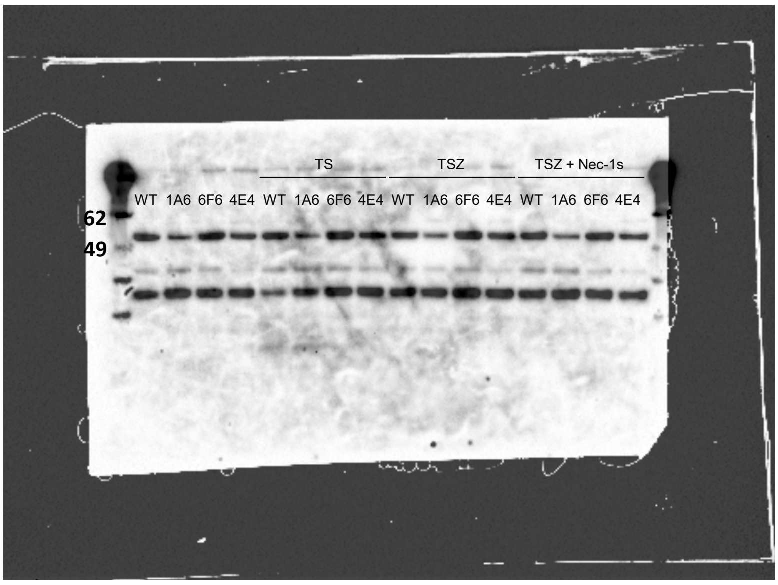

anti-cleaved caspase 3 (Asp175) [5A1E]
+ goat anti-rabbit (ab205718)

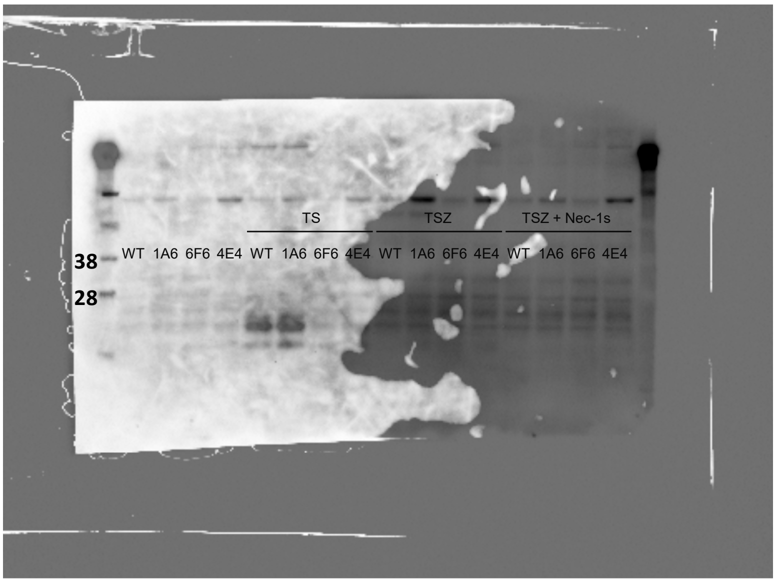

anti-caspase-3 (#9662) + goat anti-rabbit (ab205718)

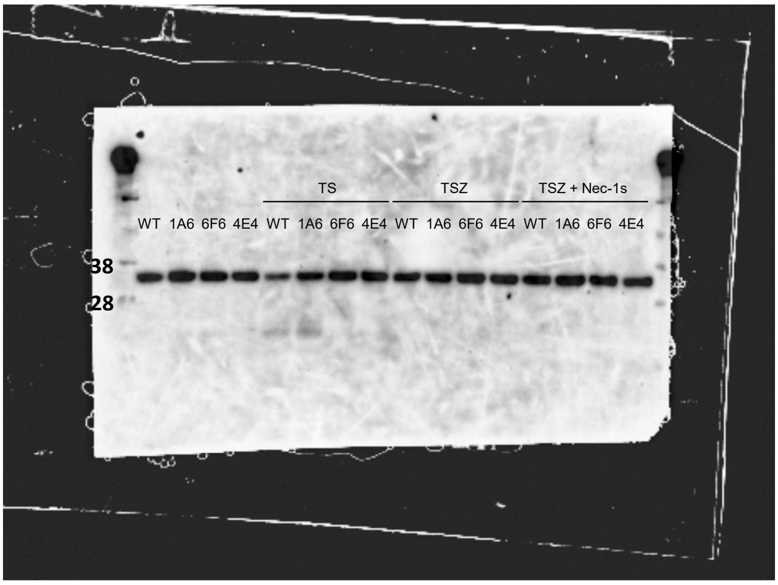

anti-ADA2 (ab288296) + goat anti-rabbit (ab205718)

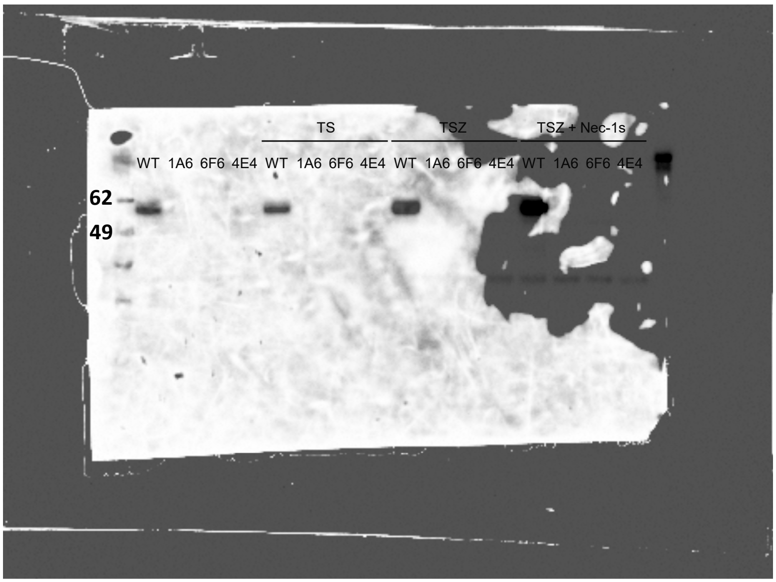

anti-β-actin (AC-15) + goat anti-mouse (#71045)

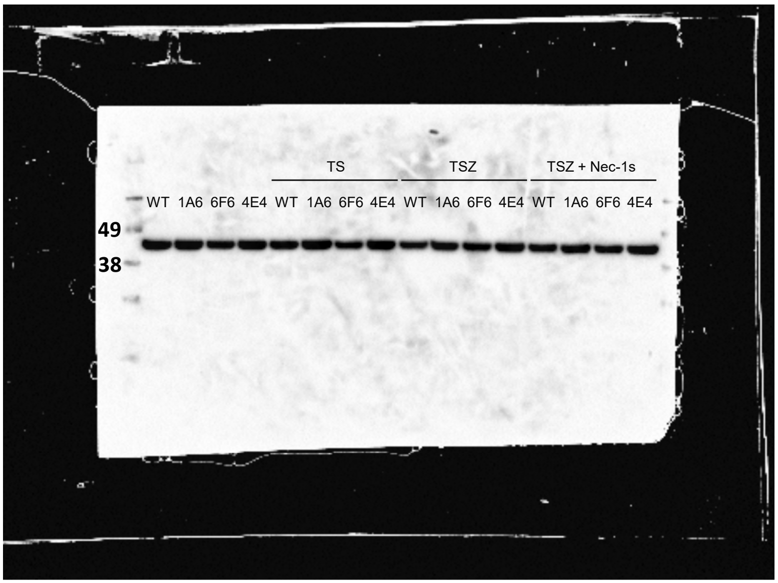

Figure S2C

anti-caspase-3 (#9662) + goat anti-rabbit (ab205718)

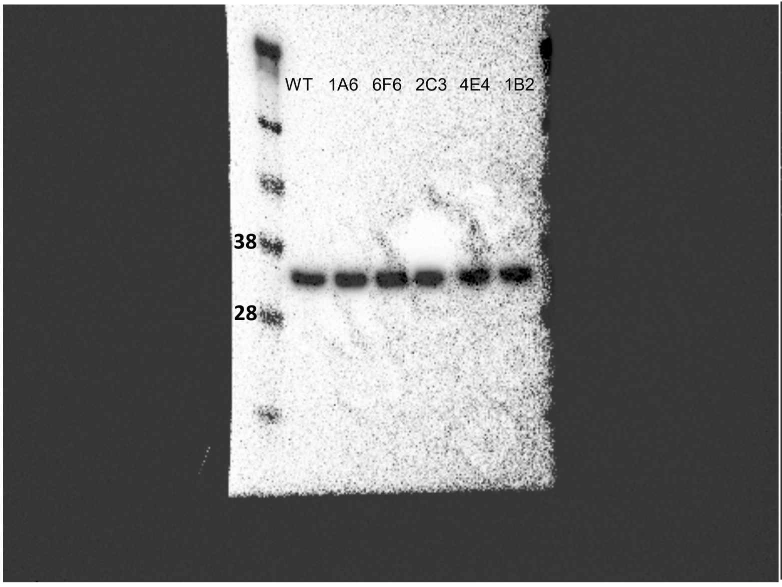

anti-MLKL [D2I6N] + goat anti-rabbit (ab205718)

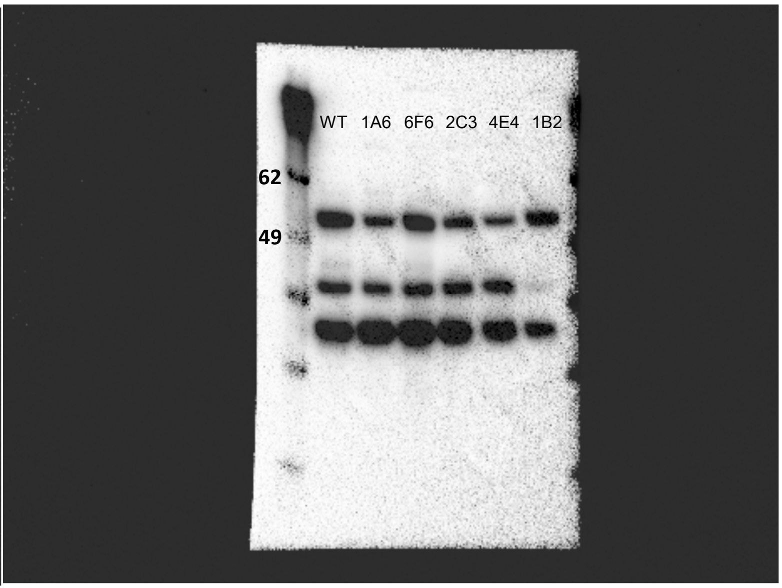

anti-ADA2 (ab288296) + goat anti-rabbit (ab205718)

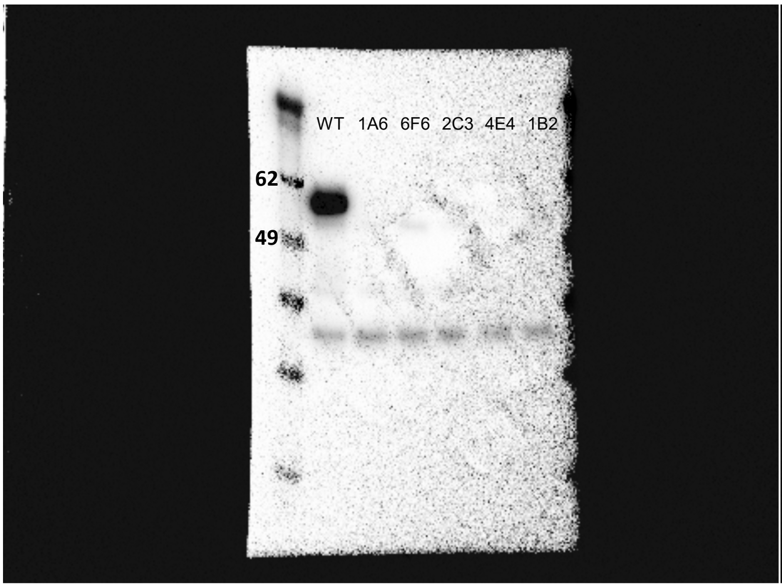

anti-β-actin (AC-15) + goat anti-mouse (#71045)

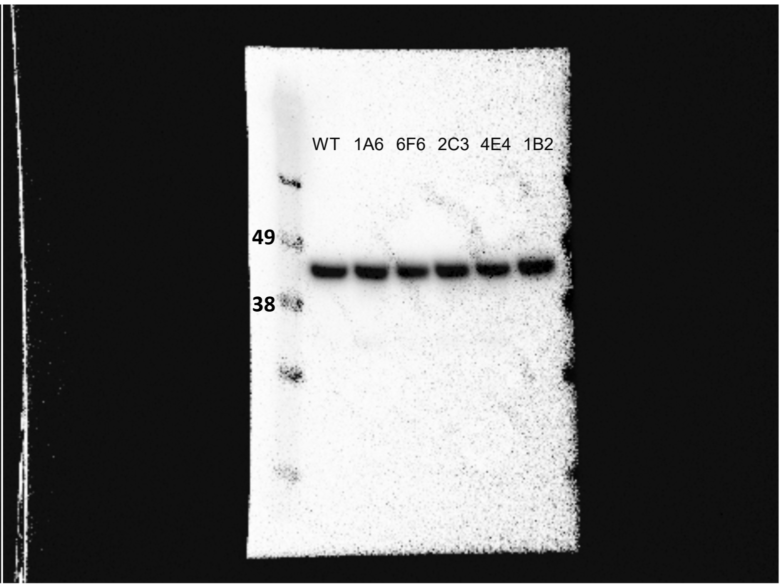

Figure S2C

anti-pMLKL (S358) [D6H3V] + goat anti-rabbit (ab205718)

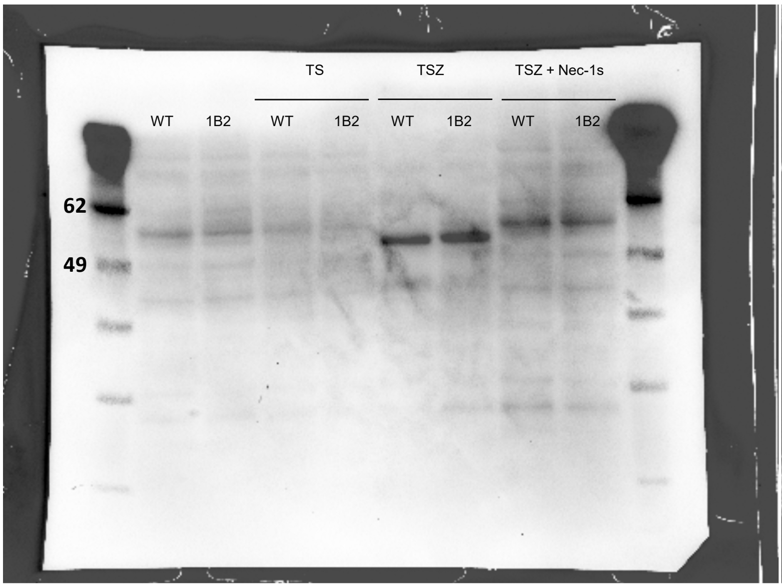

anti-MLKL [D2I6N] + goat anti-rabbit (ab205718)

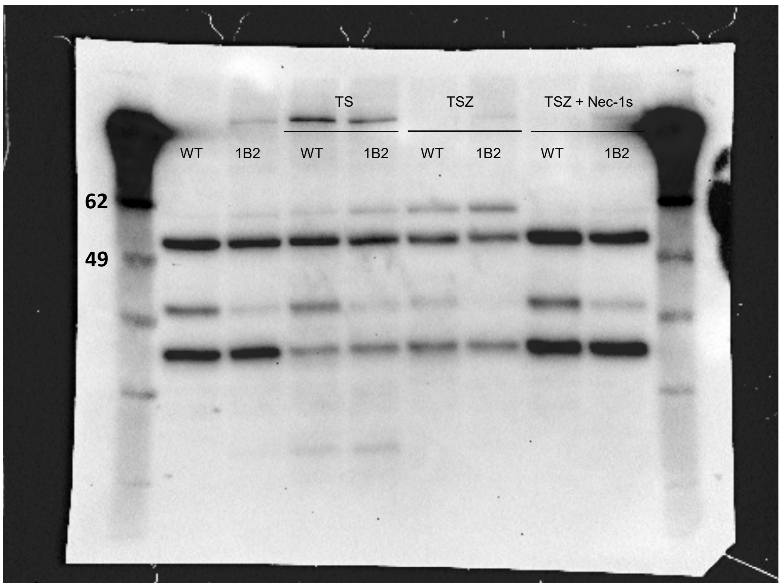

anti-cleaved caspase 3 (Asp175) [5A1E]
+ goat anti-rabbit (ab205718)

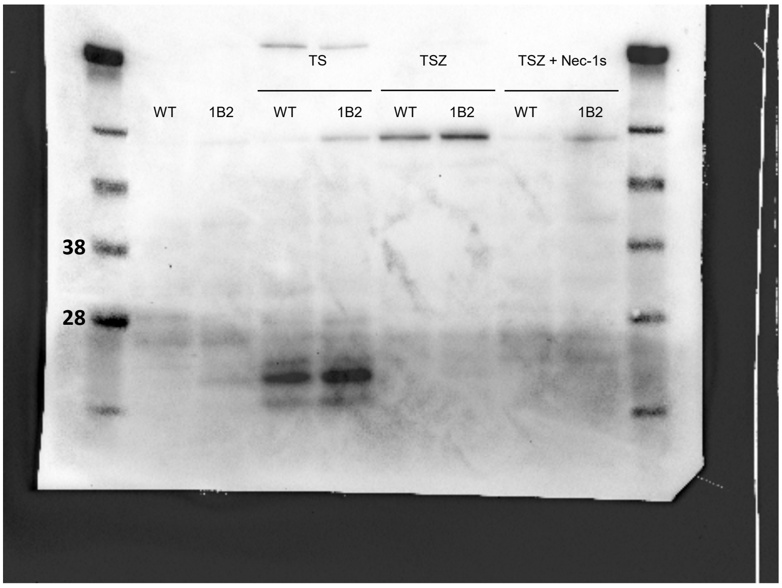

anti-caspase-3 (#9662) + goat anti-rabbit (ab205718)

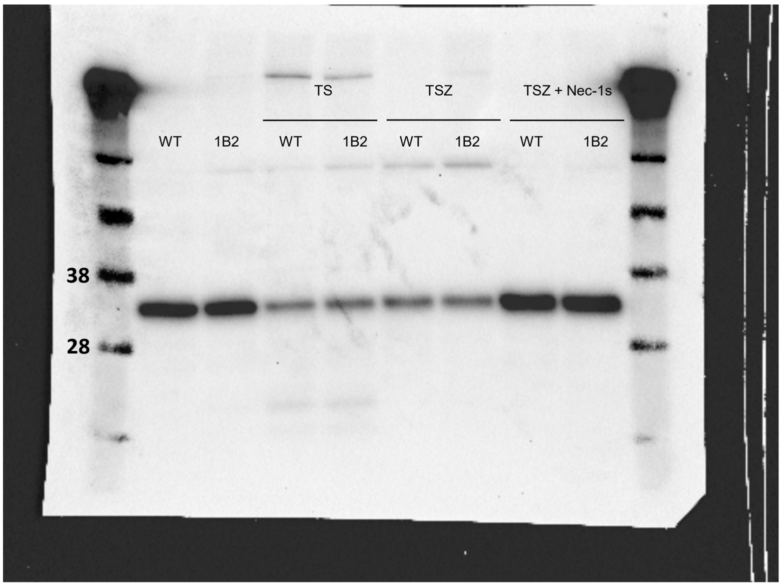

anti-ADA2 (ab288296) + goat anti-rabbit (ab205718)

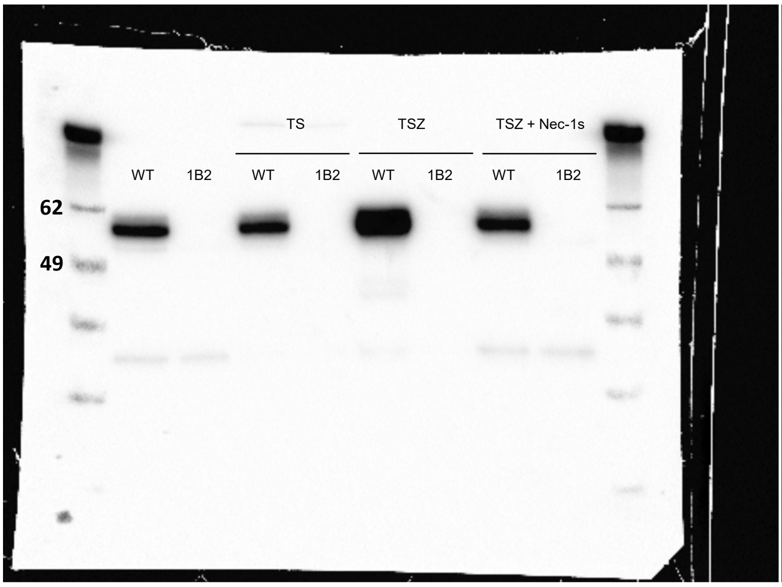

anti-β-actin (AC-15) + goat anti-mouse (#71045)

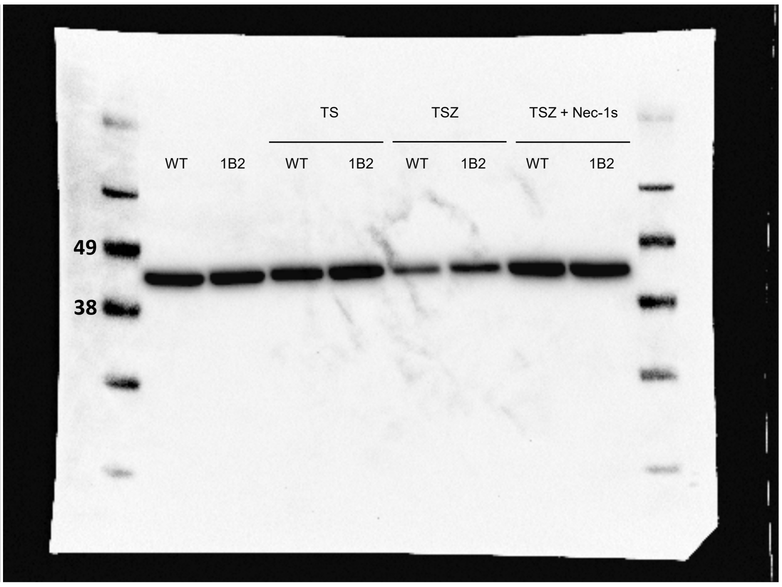

Figure S4

anti-ADA2 (ab288296) + goat anti-rabbit (ab205718)

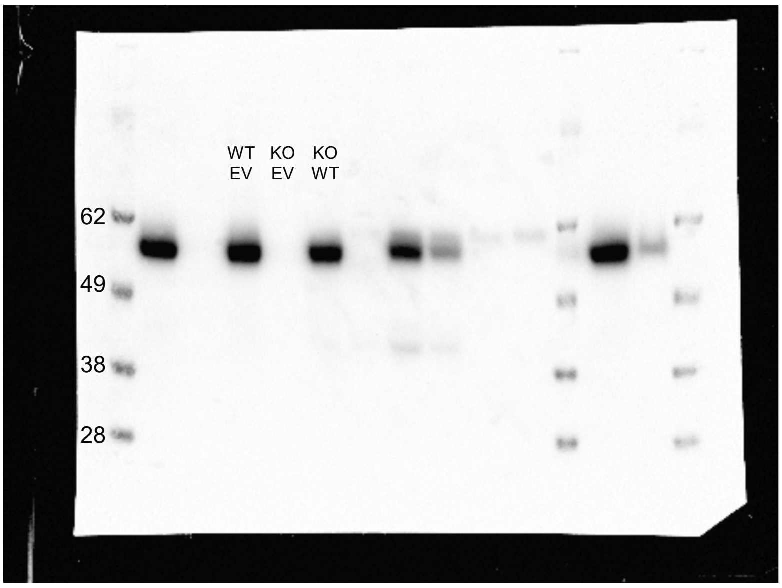

anti-β-actin (AC-15) + goat anti-mouse (#71045)

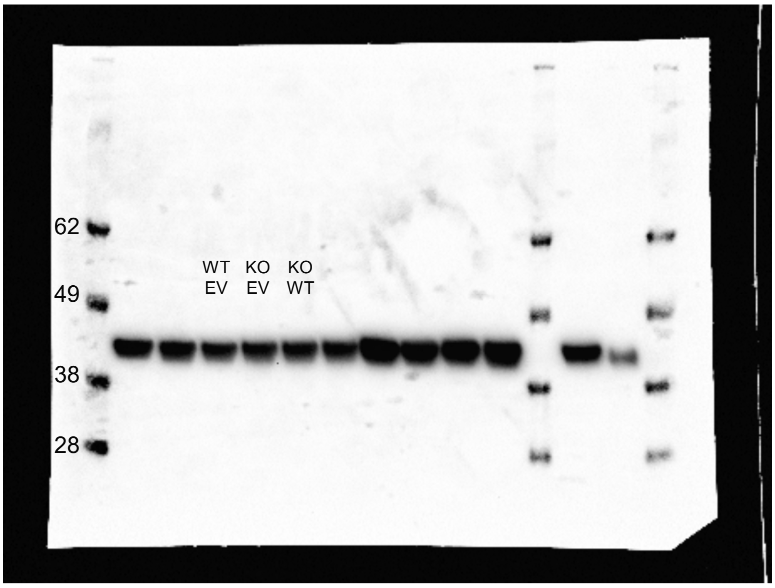

Supplement: Supplementary file 2 — Uncropped blots [file 41420_2026_3027_MOESM2_ESM.pdf]
